# Multipronged dental analyses reveal dietary differences in last foragers and first farmers at Grotta Continenza, central Italy (15,500–7000 BP)

**DOI:** 10.1038/s41598-021-82401-2

**Published:** 2021-02-19

**Authors:** Alessia Nava, Elena Fiorin, Andrea Zupancich, Marialetizia Carra, Claudio Ottoni, Gabriele Di Carlo, Iole Vozza, Orlando Brugnoletti, Francesca Alhaique, Renata Grifoni Cremonesi, Alfredo Coppa, Luca Bondioli, Dušan Borić, Emanuela Cristiani

**Affiliations:** 1 Department of Maxillo-Facial Sciences, DANTE - Diet and Ancient Technology Laboratory, Sapienza University of Rome, Rome, Italy; 2 Skeletal Biology Research Centre, School of Anthropology and Conservation, University of Kent, Canterbury, UK; 3 Department of Maxillo-Facial Sciences, Sapienza University of Rome, Rome, Italy; 4 Bioarchaeology Service, Museum of Civilizations, Rome, Italy; 5 Department of Archaeological Science, University of Pisa, Pisa, Italy; 6 Department of Environmental Biology, Sapienza University of Rome, Rome, Italy; 7 Department of Cultural Heritage, University of Padua, Padua, Italy; 8 The Italian Academy for Advanced Studies in America, Columbia University, New York, USA; 9 Department of Genetics, Harvard Medical School, Harvard University, Cambridge, USA; 10 Department of Evolutionary Anthropology, University of Vienna, Vienna, Austria

**Keywords:** Archaeology, Archaeology

## Abstract

This paper provides results from a suite of analyses made on human dental material from the Late Palaeolithic to Neolithic strata of the cave site of Grotta Continenza situated in the Fucino Basin of the Abruzzo region of central Italy. The available human remains from this site provide a unique possibility to study ways in which forager versus farmer lifeways affected human odonto-skeletal remains. The main aim of our study is to understand palaeodietary patterns and their changes over time as reflected in teeth. These analyses involve a review of metrics and oral pathologies, micro-fossils preserved in the mineralized dental plaque, macrowear, and buccal microwear. Our results suggest that these complementary approaches support the assumption about a critical change in dental conditions and status with the introduction of Neolithic foodstuff and habits. However, we warn that different methodologies applied here provide data at different scales of resolution for detecting such changes and a multipronged approach to the study of dental collections is needed for a more comprehensive and nuanced understanding of diachronic changes.

## Introduction

The transition from foraging to farming was a long-lasting and nonlinear process that took place over several millennia and enfolded at different times in different parts of the world (e.g.^1–3^). While this process is clearly reflected in changes in material culture traditions, it can equally well be observed on skeletal evidence (e.g.^4–6^). Among other human remains, teeth represent the privileged anatomical segment for the application of sophisticated analytical methods. Teeth are the most durable part of the human body; mineralized tissues capable of preserving valuable information about an individual’s biological life history. As food passes through the mouth, teeth trap direct evidence of dietary practices—either through physical–chemical changes foodstuff causes in teeth (certain dental pathologies), traces of wear, and/or foods deposited in the matrix of mineralized dental plaque. Besides information on dietary practices, various physiological processes are also recorded in dental structures.

In particular, carious lesions can be informative of the consumption of highly cariogenic wild and domesticated plant foods as they involve the progressive demineralization of the mineral component of the dental tissues by acids produced from the fermentation of food particles^7^. Dental microwear analysis is commonly used to investigate shifts in dietary habits in past human populations^8–12^. Foodstuffs chewing causes microscopic scratching and pitting on the dental surfaces due to enamel's composition, which includes particles such as silica phytoliths or exogenous grits. Variations in these microscopic wear patterns could reflect long-term trends in dietary habits^13^, given the diverse properties of foodstuffs^14^, as well as the evolution of technologies related to food processing. Microwear patterns in well-preserved enamel with no or minimal diagenetic *post-mortem* alterations can also be related to food exploitation strategies, tool technology, and the techniques used in food processing^8,12,15^. Lastly, foodstuff can be trapped in dental plaque along with microparticles generated by daily life activities or environmental conditions. Once inhaled or trapped in the plaque, such materials can be preserved in the dental calculus, i.e. the deposits formed through the mineralization of the plaque biofilm, which can survive over millennia protected from modern contamination (e.g.^16–18^).

**Figure 1:**
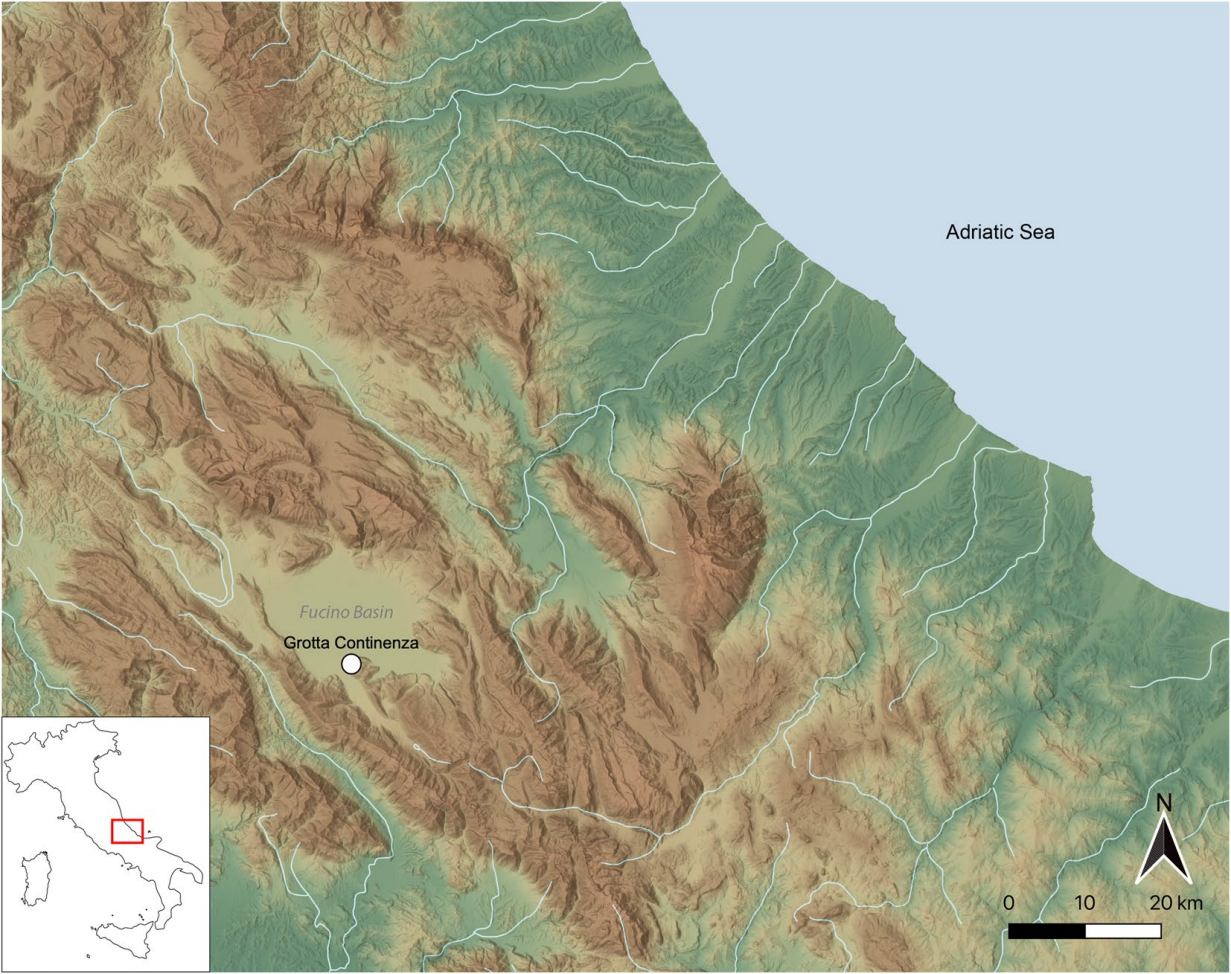
Location of Grotta Continenza in the Fucino Basin (Abruzzo).

Analysing trends in dietary patterns across major cultural and technological transitions is pivotal for reconstructions of biological and techno-cultural adaptations of human groups. It is a rare occasion to be able to observe these changes in a prehistoric series from a single site. In this paper, we utilize teeth to study changes in palaeodiet in central Italy during the forager-farmer interval by focusing on a unique cave sequence found at the site of Grotta Continenza, situated in the Fucino Basin of the Abruzzo region of central Italy (Fig. 1). The site has yielded numerous human remains spanning the end of the Palaeolithic (from ca. 15,500 cal BP) until the Roman period^19–21^. Our hypothesis is that major palaeodietary changes should be identifiable among the different cultural and chronostratigraphic units of Grotta Continenza.

In order to test our hypothesis, we utilize three main analytical proxies for studying diet at this site: (1) dental metric and oral pathologies, (2) buccal microwear and (3) micro-fossils preserved in the mineralized dental plaque. Methodologically, we are interested in understanding to what extent can these different types of analysis be complementary when applied to the same dental material in disclosing differences in dietary intake between foragers and farmers. At this stage in our research, this is done without relying on the frequently used methodology for discerning palaeodietary patterns by analysing stable isotopes, which is the focus of an ongoing project^22^. Our results from Grotta Continenza indicate differences between the last foragers and first farmers in patterns of the presence of caries, buccal microwear, and in the selection of plant foods, supporting a technological shift in the food exploitation and plant processing techniques brought about by the onset of the Neolithic.

## The Grotta Continenza site

Grotta Continenza opens onto the northern slopes of Mount Labrone, 710 m asl and 43 m above the southern limits of the present-day Fucino Basin (Fig. 1). This region was previously occupied by a palaeolake (Lago Fucino), the level of which oscillated repeatedly during the Late Pleistocene and Early Holocene^23^. The site includes a rock-shelter about 20 m wide and 7–8 m deep, representing the main part of the cave, and an inner space, the proper cave, about 8 by 8 m wide. While no evidence of palaeolake shoreline (e.g. primary lake sediments) were identified inside the cave, sediments from the site have been included in the altitude belt (between 685 and 715 m asl) that documents the highest levels of the Fucino Lake^23^.

**Figure 2:**
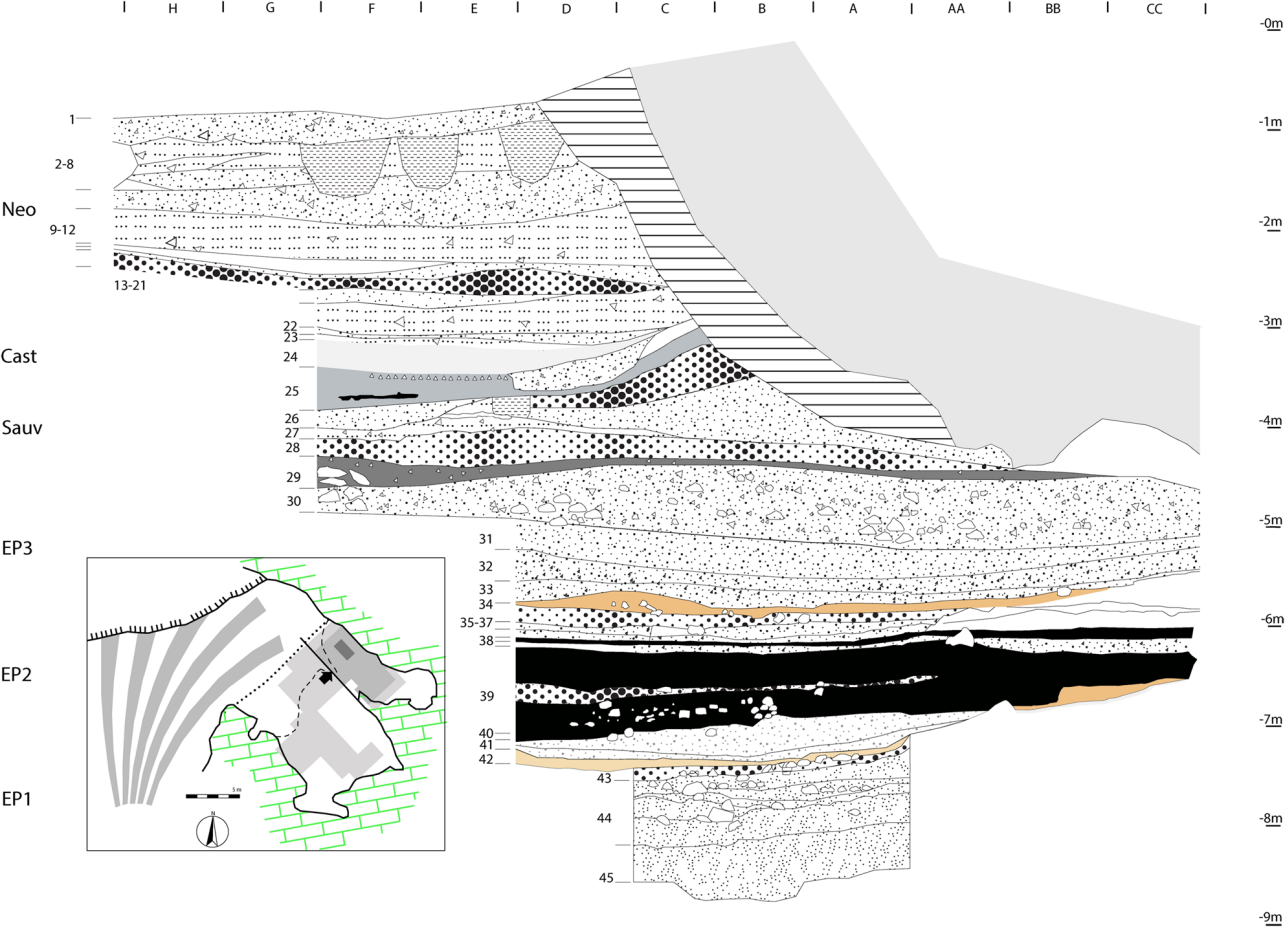
Longitudinal stratigraphic profile of Grotta Continenza. Retraced and adapted after Boschian et al. 2017.

Systematic excavations, carried out from 1978 to 2013, revealed a 9-m-deep stratigraphic sequence^21,24–27^, with early prehistoric strata documenting a continuous use of the site from the Last Glacial (ca. 15,500 cal BP) to the Early Holocene (ca. 7000 cal BP) (Fig. 2). A recent study by Boschian et al.^28^ provides a comprehensive overview of the stratigraphy and dating of the sequence at Grotta Continenza, suggesting the following groupings of cuttings into several main chrono-cultural units (from top to bottom): Roman period, Bronze Age, Eneolithic (cuttings 1–2), Middle and Early Neolithic (cuttings 2–22), Late Mesolithic/Castelnovian (cuttings 23–24), Early Mesolithic/Sauveterrian (cuttings 25–28), and the final phases of the Late Upper Palaeolithic/Epigravettian (cuttings 29–48). This division was based on both the characteristics of the associated material culture found in each of the cuttings as well as a series of 27 radiocarbon measurements (17 conventional and 10 direct acceletator mass spectrometry [AMS] dates) available at the time.

Since the publication of the study of Boschian et al.^28^, another 11 AMS dates have most recently become available for human remains analysed for ancient DNA (aDNA) (^29^: Tables S2–3). Of 11 dates, three fall in the assumed duration of the Early Mesolithic Sauveterrian phase (UCI-198583, UCI-198579, UCI-198584), one in the assumed duration of the final Mesolithic Castelnovian phase (UCI-198574), five in the assumed duration of the Early Neolithic (UCI-198575, UCI-198580, UCI-198582, UCI-198581, UCI-213625), and two in the final phases of the Italian Eneolithic (UCI-198576, UCI-198577). While there is an overall agreement between the provenance of the human remains on which these dates were made and attributions of cuttings to chrono-cultural units suggested by Boschian et al.^28^, there are also several new insights provided by the new dates. They suggest that cuttings 21–22 and 14–15, assumed to have corresponded with the earliest Neolithic, contain some residual Mesolithic material of Sauveterrian and Castelnovian provenance respectively. On the other hand, there is also evidence of Eneolithic intrusions that deposited human remains dated by UCI-198577 in cutting 10. In order to alleviate somewhat possible issues with the chronological attribution of some human remains analysed in this study, especially for the remains with no information on the cutting from which they originate, we report here for the first time two new AMS radiocarbon measurements for individuals GC14 and GC44. OxA-39688 dates individual GC14 in 9351 ± 32 BP with the calibrated range of 10,675–10,440 cal BP at 95% confidence, falling into the Early Mesolithic, Sauveterrian phase of occupation. OxA-39685 dates individual GC44 in 6837 ± 26 BP with the obtained calibrated range of 7715–7610 cal BP at 95% confidence, falling into the Early Neolithic phase of occupation.

Over more than 8500 years, Grotta Continenza was repeatedly used as a dwelling and a funerary place. The stratigraphic sequence encompasses a total of 48 cuttings. Five hearths and abundant lithic and faunal remains indicate an intense occupation of the site with its exclusive use for daily life activities in the earlier phases of the Late Epigravettian (phases EP1 and EP2, cuttings 48–35, ca. 15,690–13,100 cal BP). Fireplaces are also documented in the later phase of the Late Epigravettian (EP3, cuttings 34–30, ca. 12,400–11,200 cal BP) when seven individuals were found buried in cuttings 34–33 and 32–31, indicating the initial use of the site for funerary practices^20,21^.

During the Early Mesolithic, Sauveterrian phases (ca. 11,200–10,500 cal BP), the outer rock-shelter and the entrance to the inner cave continued to be used as a dwelling place but also contained a number of disarticulated human remains. The recovery of numerous disarticulated human remains, as well as at least nine disarticulated individuals and one articulated inhumation burial in the Late Mesolithic, Castelnovian layers 23–24 (ca. 8500–7800 cal BP), indicate that the site was used for funerary practices throughout the Mesolithic.

In the Neolithic (layers 2–22, ca. 7600–6900 cal BP), dwelling activities were limited to the outer space, while the inner part of the cave was destined for funerary practices only. In particular, disarticulated remains of at least 45 individuals were recovered, including 18 juveniles. No burial pits were identified during the excavations, hence corpses might have been deposed directly on the ground, without specific arrangements. The only exception is represented by an old woman whose corpse was associated with two vessels containing the incinerated remains of a 4- and 8-year-old individuals^21^.

## Materials and methods

All the necessary permissions for carrying out the study of the archaeological teeth from Grotta Continenza have been obtained from the “Soprintendenza Archeologia, Belle Arti e Paesaggio dell'Abruzzo” ahead of our analysis. Moreover, the analyses discussed in this article were all carried out in accordance with local relevant guidelines and regulations.

Examined dental material consists of a total of 140 human teeth, pertaining all tooth classes and both arches (Supplementary Table S1). Among these, 88 are isolated teeth, while the remaining are *in situ* in mandibles (7 mandibles, 19 teeth) or maxillae (8 maxillae, 33 teeth). The original archaeological labels of the human specimens, which changed in the course of many excavation campaigns, were unified in a single coding system and are reported in Supplementary Table S2.

The chronological attribution of the analysed dental remains relies on previously published information about stratigraphic units^28^ from which these remains originate as well as on two new AMS radiocarbon dates on these remains reported here for the first time (see above). Due to the issue of a limited sample size and in order to enable meaningful comparison between different periods, the dental remains from Grotta Continenza are grouped into three main chronological periods: Epigravettian (EP, cuttings 48–30, n = 27), Mesolithic, encompassing both the Sauveterrian and Castelnovian levels (MES, cuttings 23–29, n = 66), and Neolithic (NEO, cuttings 3–22, n = 47).

Based on *in situ* teeth, the Minimum Number of Individuals (MNI) is eight (three maxillae are associated with three mandibles). Among the remaining 88 teeth, the estimated MNI is 44, based on the stratigraphic association, wear stages, and morphology, thus resulting in a MNI total of 52.

No attempt was made to determine the age at death of the individuals because the sample comprises mostly isolated teeth, thus preventing a reliable age estimation. Similarly, the fragmentary nature of the maxillae and mandibles does not allow for a consistent determination of sex. Amelogenin-based sex determination^30,31^ was not performed here given the destructive nature of the methodology. However, previous studies seem to indicate that buccal microwear patterns are not affected by individual sex or age-at-death^9,15,32,33^. Similarly, no other destructive analyses, such as C, N and O isotope of dentine collagen^34^, have been carried out on this dental series. The absence of age-at-death and sex distributions for the specimens weakens the reliability of the results of the analysis of age-related traits, like macrowear and oral pathologies prevalence.

### Metric analysis, oral pathologies, macrowear, and enamel hypoplasia

The whole dental sample was scored for the presence of oral pathologies, enamel growth defects, and extramasticatory modifications. Caries, abscesses, and alveolar resorption were recorded for degree and localization according to^35^. All teeth adequately preserved for macroscopic examination were scored for the presence, localization (crown, neck, root and mesial, distal, buccal, lingual, occlusal) and degree of caries (1 to 4, from superficial to the complete destruction of the crown) following a visual inspection by means of a magnifying glass. Linear enamel hypoplasia (LEH) was scored by macroscopic examination as present, absent or not observable. The high wear levels affecting the dental series inhibited an estimate of their age of occurrence^36,37^. Extramasticatory wear and chipping were recorded macroscopically following^35^. Bucco-lingual (BL) and mesio-distal (MD) diameters (in unworn or moderately worn teeth) were recorded according to^38^ using a digital caliper. MD diameters were taken as the maximum width of the tooth crown in the mesiodistal plane, BL diameters were taken as the widest point of the tooth crown in the buccolingual plane, perpendicular to the MD. The crown area was estimated by multiplying the BL and the MD diameters. The degree of wear was recorded for each tooth according to^39,40^. Macrowear was recorded along an eight-point scale based on the amount of exposed dentine.

### Buccal microwear analysis

Microwear analysis of the vestibular/buccal aspect of premolars and molars^15^ was adopted to discern dietary patterns at Grotta Continenza. Only teeth showing macroscopically well-preserved buccal enamel surfaces without *post-mortem* physical or chemical alterations were selected for buccal microwear analysis. A control over post-depositional processes, possibly affecting the tooth surface, was done by scoring for micro-striations the interstitial facets of all teeth. The teeth showing microwear-like patterns of the interstitial facet were excluded from the analysis^8^. The analysis was performed on a total of 57 selected teeth (premolars and molars preserving at least two-thirds of the buccal aspect), attributed to unique individuals. This attribution was made on the basis of the specimens’ location (cutting and square). For those elements found in the same stratigraphic context, an evaluation of the degree of wear, colour, and presence of interproximal facets allowed us to select only the teeth securely attributed to different individuals.

Buccal microwear analysis was done on high-resolution replicas of the buccal surface. Prior to the moulding procedure, enamel surfaces were cleaned with acetone and a cotton swab. High-resolution moulds of buccal enamel surfaces and interstitial facets were obtained with a light body polyvinylsiloxane silicone (Provil^®^ Novo Light C.D.2, Heraeus Kulzer GmbH), and transparent casts were produced using bi-component epoxy resin (EpoThin 2, Buehler Ltd) (see^41^ for a detailed explanation of the methodology).

The buccal microwear pattern analysis of the selected premolar and molar teeth was performed on two-dimensional images of a square region of interest (ROI) of the replicas of the crown surface under advanced multifocal stereomicroscopy (Zeiss Axio Zoom V16) with a magnification of 80X  using the Zeiss ZEN Core (version 2.5) software. Each ROI was located on the medial third of the buccal surface, free of dental pathologies, enamel defects, or diagenetic alterations, and covered an area of 0.56 mm^2^ (square ROI of 0.748 times 0.748 mm). This 0.748 mm side length of the ROI was chosen in order to be easily compared to the data reported in literature^8^.

Data collection of the buccal microwear features was performed using a Delphi implementation (version XE7, Embarcadero Technologies) of the MicroWeaR package^42^. MicroWeaR Delphi allows for the image pre-processing with image enhancement routines, high-pass, and directional kernel-based filtering, calibration of the image, ROI definition, and tracing of the scratches (Supplementary Fig. S1). After the manual tracing of the scratches, the software generates a total of 15 variables describing the patterning of the scratches for each ROI, following^8^ (Supplementary Table S3).

The statistical package R (ver. 4.0.2)^43^ was used for all statistical computations and generation of graphs. Robust Principal Component Analysis (rPCA^44,45^, resistant to outliers in the data, implemented in the “rrcov” package of R^46^), was carried out based on the 15 variables reported in Supplementary Table S3.

### Dental calculus

Dental calculus was collected from a total of 21 individuals, attributed to the Epigravettian (n = 7), Sauveterrian (n = 4), Castelnovian (n = 4), and Neolithic (n = 6) on the basis of their stratigraphic provenance and/or direct AMS dating of human remains. We conceived the sampling strategy so to maximize the usefulness of the destructive analysis of ancient mineralized plaque, which represents a valuable evidence of biomolecular, protein, and aDNA data about ancient health and diet as well as microbial and bacterial data about pathogens. Accordingly, a portion of dental calculus deposit was left on the teeth for future analysis.

Dental calculus was removed from the tooth using a metal dental scaler and immediately stored in sterile plastic tubes (0.5 ml) ^78^. The weight of the mineralized plaque removed from the individuals ranged from 0.6 to 6.7 mg (Supplementary Table S4). Mineralized plaque sampled from Grotta Continenza teeth has undergone two different studies: (a) the metagenomic analysis through shotgun sequencing aimed at reconstructing oral bacterial aDNA. The results of this analysis have recently been submitted for publication elsewhere ^79^; (b) the micro-debris analysis aimed at identifying possible residues of foods or evidence for the extramasticatory use of teeth/mouth.

With regards to the study of microfossils, the decontamination and the extraction procedures were conducted at the DANTE—Diet and Ancient Technology Laboratory of Sapienza University of Rome according to standard protocols as described by^47^. To avoid modern contamination, several standard procedures were followed (e.g. starch-free gloves were worn at all times and instruments were cleaned and changed for each sample). In addition, samples of contaminants possibly present on the instruments (e.g. bristles) and on work surfaces were checked under the microscope as well as the contaminated soil washed away from the archaeological specimens. The extraction and mounting of ancient samples occurred in a laboratory not connected to modern botanical work and under strict environmental monitoring.

The first step of the decontamination process consisted in the removal of the mineralised soil adhering to the surface of the calculus. Samples were cleaned using tweezers to hold the sample and a fine sterile acupuncture needle to gently scrap off the soil attached to the external layer of the plaque. The procedure was performed using drops of 0.5 M Hydrochloric acid to dissolve the mineralised flecks of soil and ultrapure water to wash and remove the contaminants. In the case of very small samples, a fine paint brush with nylon bristles was employed instead of the acupuncture needle. The decontamination procedure was carried out under a stereomicroscope at a magnification of up to 100X.

Once the surface was cleaned, samples were washed in ultrapure water up to three times in order to remove any trace of sediment. The contaminated soil was stored for the purpose of checking for a possible cross contamination. Thereafter, the calculus was dissolved in a solution of 0.5 M Hydrochloric acid and subsequently mounted on slides using a solution of 50:50 glycerol and ultrapure water. Examination of the micro-debris embedded in the calculus matrix was performed using a Zeiss Imager2 cross polarised microscope with magnifications up to 630X. The modern reference collection of plants native to the Mediterranean and Balkan regions, and Europe stored at DANTE Laboratory and the previously published literature were employed in comparisons.

## Results

### Metric analysis

**Figure 3:**
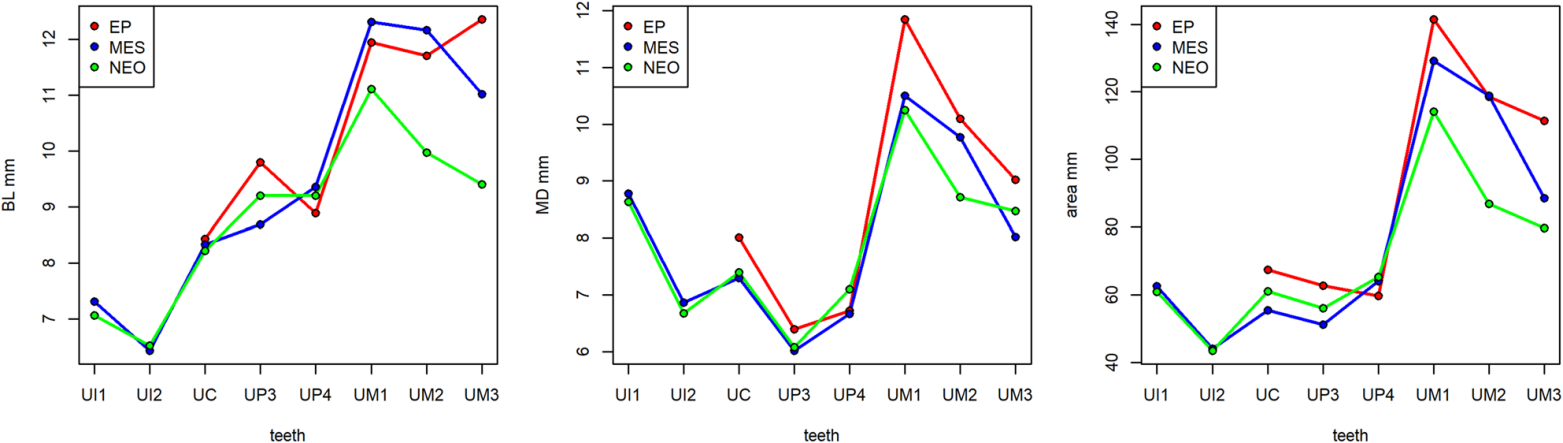
Variation of the mean crown diameters and area of the upper dentition through the periods. (**a**) Trend through time of the mean BL diameters; (**b**) Trend through time of the mean MD diameters, (**c**) Trend through time of the mean of the areas. EP = Late Epigravettian (n = 15), MES = Mesolithic (Sauveterrian and Castelnovian, n = 38), NEO = Early and Middle Neolithic (n = 26).

The metric analysis was performed on a subset of 79 teeth, paying attention to exclude one of the possibly present antimeres from the same individual/unit. Base statistical parameters of the BL and MD variables are presented in Supplementary Table S5. Figure 3 shows trends over time of the mean BL diameters (Fig. 3a), of the mean MD diameters (Fig. 3b), and the mean of the areas (Fig. 3c) across the different tooth classes in the upper dentition.

For the upper molars, the general trend of sizes is Epigravettian > Mesolithic > Neolithic, with the exception of the MD diameter of the third molar. Both incisors show no differences between the Mesolithic and the Neolithic, while not enough data are available for the Epigravettian. For the canines, the MD diameter is larger in the Epigravettian group. Among premolars, the smallest is the third premolar from the Mesolithic sample (Fig. 3c). As an exception from the general trend, the fourth premolar of the Neolithic group exceeds the size of both Epigravettian and Mesolithic fourth premolars. The lower dentition (Supplementary Fig. S2), even if represented by a limited number of specimens, approximately follows the same trend of the upper dentition, apart from the fourth premolar, which is smaller in the Neolithic sample. Moreover, the means of the mandibular first molars tend to be larger in the Mesolithic sample compared to the Epigravettian sample.

### Macrowear

The results of the analysis of macrowear patterns are reported in Supplementary Fig. S3. The Epigravettian sample shows a higher mean degree of dental wear, particularly in the higher grades, while the Neolithic group shows the lower degrees of macrowear. The difference is statistically significant (Pearson Chi-squared test with simulated p-value based on 10,000 replicates, chi-squared = 25.1, p = 0.03).

### Dental linear enamel hypoplasia

LEH is present in 35.6% of the observable teeth. It is rarely multiple (1.9%). The generalised high wear degree inhibits the scoring of the hypoplastic defects in mid to occlusal portions of the crowns. Indeed, the association between the presence of hypoplasia and wear degrees is statistically significant (chi-squared test with simulated p-value based on 10,000 replicates, chi-squared = 26.231, p < 0.001, Cramer’s V of association = 0.5). The distribution of LEH among the periods indicates a reduced proportion of affected teeth in the Epigravettian (EP = 3/19 = 15%, MES = 20/49 = 41%, NEO = 14/33 = 42%). However, the lower prevalence during the EP is strongly affected by the high macrowear observed in this period.

### Oral pathologies

Carious lesions are rare in the sample (16% of the observable crowns, n = 17/104), and the Neolithic group is only slightly more affected than the others (EP = 3/23 = 13% of the EP scored teeth, MES = 7/47 = 15% of the MES scored teeth, NEO = 7/34 = 21% of the NEO scored teeth). Incisors are always unaffected by caries (0/25), a single case is observable among canines (1/15 = 7%), three among premolars (3/23 = 13%), and thirteen among molars (13/41 = 32%). The analysis of degree and localization of caries shows no differences among the cultural groupings, possibly due to the small number of teeth affected by caries. Regarding oral pathologies affecting the bone, almost all of the examened alveoli (96%) evidence alveolar resorption, while only one case of an abscess is found in the Neolithic sample.

### Extramasticatory wear and chipping

Extramasticatory wear of anterior and premolar teeth was observed in 10.6% of the MES teeth and in 6.0% of the NEO teeth. Chipping is less frequent and rarely involves the occlusal surface (EP = 2/22 = 9%, MES = 2/43 = 5%, NEO = 1/31 = 3%).

### Buccal microwear analysis

Almost all of the analysed teeth presented a well-preserved enamel buccal surface at the microscopic level. Only two among the selected teeth presented buccal-like microwear scratches on the interstitial facets as a consequence of post-depositional damage. Six more teeth showed a poor state of preservation of the buccal surface at the microscopic level. Those teeth were excluded from the analysis. Therefore, the analysis was performed on a total of 49 selected premolars and molars dated to the Late Palaeolithic (n = 13), Mesolithic (n = 21), and Neolithic (n = 13) on the basis of their stratigraphic provenance and/or direct AMS dating of human remains. Two more teeth without a closer chronological assessment were included in the rPCA analysis in order to increase the sample size.

The buccal enamel surface showed scratches of various length and frequency with a main vertical orientation, followed by the two oblique (MD and DM) directions. The less represented orientation is the horizontal one (Table 1).

**Table 1:** Density by orientation, all teeth.

Orientation	Mean	sd	IQR	Min	Median	Max	n
All	121.5	46.8	70	33	111	225	49
Vertical	39.6	24.0	26	5	36	140	49
Horizontal	22.8	13.1	20	2	19	54	49
Oblique MD	27.6	22.1	20	0	25	87	49
Oblique DM	31.4	17.8	23	5	28	77	49

*IQR* = interquartile range.

The means of the density of scratches are not significantly different among tooth classes (analysis of variance with 4 degrees of freedom, F value = 1.044, p = 0.395) as shown in Supplementary Fig. S4.

Similarly, the means of the length of the scratches are not significantly different among tooth classes (analysis of variance with 4 degrees of freedom, F value = 1.445, p = 0.235) as shown in Supplementary Fig. S5.

Supplementary Fig. S6 reports the box and whisker plot of the number of scratches by chronological period. The analysis of variance shows a significant difference between the means of the chronological periods (2 and 44 degrees of freedom, F = 3.262, p = 0.0478). Tukey’s Honestly Significant Difference test reports a close to a significant difference between the mean of the Neolithic and the Mesolithic groups (p = 0.061), no differences between the Mesolithic and the Epigravettian groups (p = 0.998), and between the Neolithic and the Epigravettian groups (p = 0.09).

Supplementary Fig. S7 shows that the mean length of the scratches remains constant through time, with a modest decrease in the Neolithic period (analysis of variance with 2 and 44 degrees of freedom, F = 0.589, p = 0.559).

**Figure 4:**
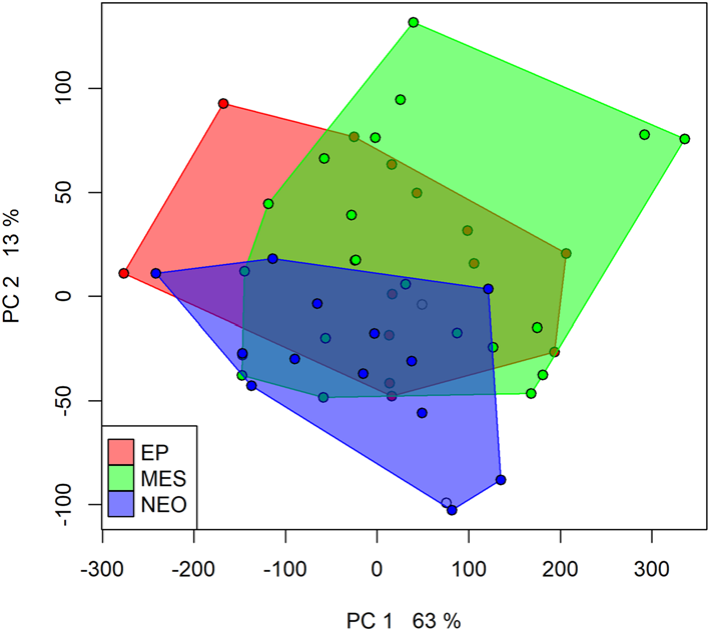
Scatterplot of the first and second principal component scores.

Figure 4 shows the scatterplot of the first two principal components, which together explain 76% of the total variability. The cluster of the Epigravettian teeth partially overlaps with the Neolithic cluster, while the Mesolithic teeth show the largest variation across the space of the components, thus suggesting a slightly more varied foodstuff exploitation and processing pattern. The component loads tend to contrast the scratch density along all directions with a mean and standard deviation of the lengths (see Supplementary Fig. S8).

**Figure 5:**
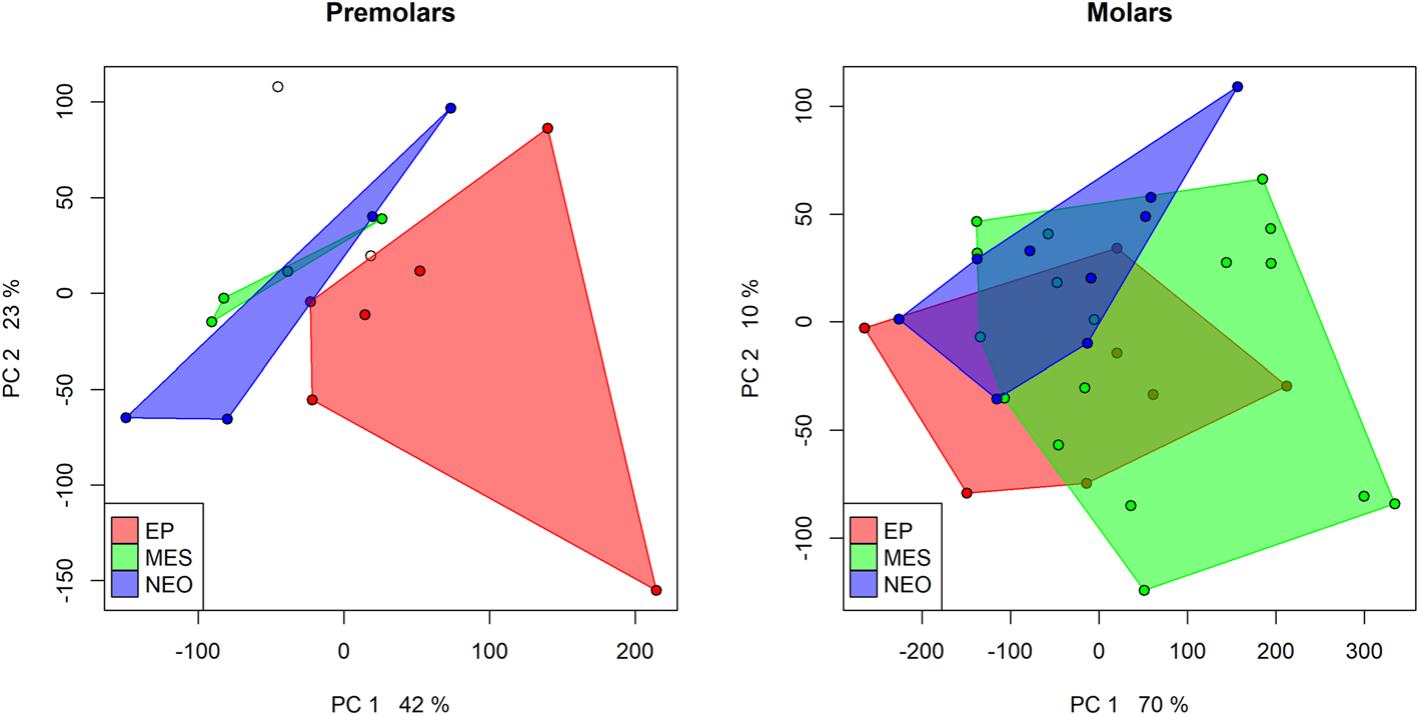
Scatterplots of the first and second principal component scores. Left panel third and fourth premolars only; Right panel molars only.

Figure 5 shows the rPCA of the premolars (Fig. 5 left panel) and molars (Fig. 5 right panel) separately. The principal component analysis of the premolars’ scratch patterns in the two first components (explaining together 65% of the total variance) separates the Neolithic teeth from the Epigravettian ones. The Mesolithic teeth are partially separated from the other groups. The rPCA of the molars in the two first components (explaining together 80% of the total variance) reproduces the pattern observed when all of the teeth are considered together.

### Dental calculus

Of 21 sampled and analysed individuals, 16 individuals/teeth yielded micro-debris embedded in dental calculus (Table 2). Plant remains from these specimens have been identified in the form of wood elements, plant fibres, charcoals, phytoliths, and starch granules. It is starch granules that provide the only reliable line of evidence that can be linked to the deliberate consumption of plant foods.

**Table 2:** Micro-debris retrieved in the dental calculus samples from Grotta Continenza.

No	Individual	Cutting	Chronology	Starch type 1 (Triticeae)	Starch type 2 (Paniceae)	Starch NI	Charcoal	OPL	Wood	S/P	AR
1	GC 9	2	Neolithic			1	15			1	
2	GC27	4	Neolithic				> 13	2	1		
3	GC 44	5	Early Neolithic (OxA-39685)	23		12				> 4	
4	GC 72	10	Neolithic				1	5	1		
5	GC 15	14–15	Neolithic				> 12	17			
6	GC 8	24	Late Mesolithic				6	4			1
7	GC 71	24	Late Mesolithic				2	1			
8	GC 28	25	Early Mesolithic		2	6	49			1	
9	GC 76	25	Early Mesolithic				4	3			
10	GC 36	25–26	Early Mesolithic		> 80	13	2				
11	GC 14		Early Mesolithic (OxA-39688)		1	1	> 14	4		1	
12	GC 58	29–30	Late Upper Palaeolithic				> 2	3			
13	GC 21	30	Late Upper Palaeolithic				1	4	1		
14	GC 61	30	Late Upper Palaeolithic					12			
15	GC 33	36	Late Upper Palaeolithic			1				> 50	
16	GC 60	–	Late Upper Palaeolithic	1	1		19	6	1		
			Total	24	> 82	38	> 140	57	4	> 7	1

Starch Type 1 (Triticeae); Starch Type 2 (Paniceae); *Starch NI* starch not identified, *OPL* other plant remains: fibres and vegetal tissues; *S/P* spores and pollen grains; *AR* animal remains; the symbol “ > ” is used when starch granules or other microbotanical fossils cannot easily be counted, for example when embedded in dental calculus.

### Starch granules

**Figure 6:**
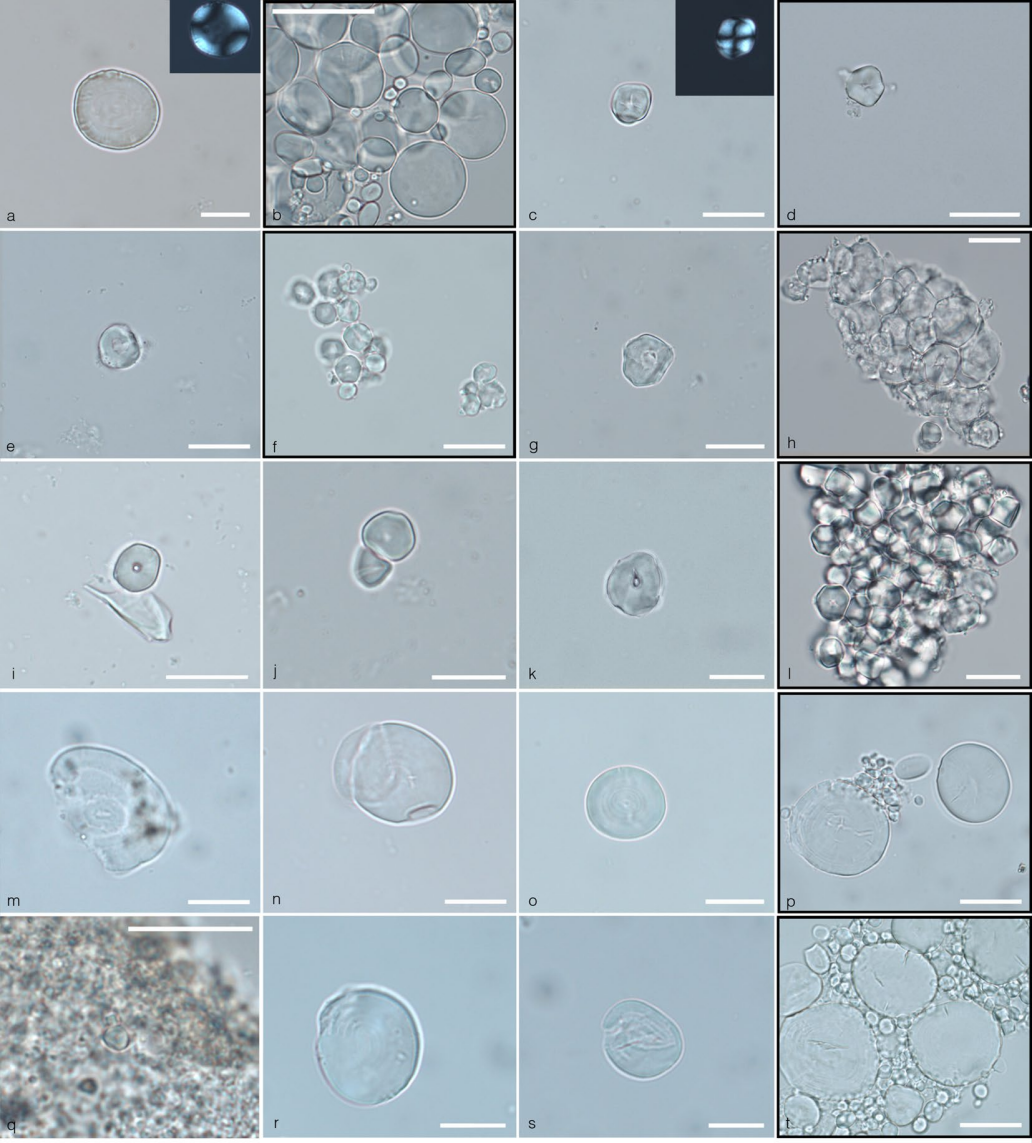
Archaeological starch granules identified in Grotta Continenza individuals and experimental reference (black-framed photos). (**a**) Starch granule from GC33 (Sauveterrian); (**b**) Large circular starch grains of *Aegilops geniculate* characterized by mesial lamellae; (**c**) Starch granule from GC 28 (Sauveterrian); (**d**) Single polyhedral starch granule of *Setaria ventricolata* characterized by a central depressed hilum, fissures and no lamellae; (**e**) Starch granule from GC36 (Sauveterrian); (**f**) Polyhedral and circular starch granules of *Echinocloa crussgalli* characterized by a central depressed hilum and no lamellae*;* (**g**) Starch granule from GC36 (Sauveterrian); (**h**) Polyhedral starch granules of Foxtail millet (*Setaria italica*) processed with a ground stone tool, characterized by a central depressed hilum and no lamellae. Notice the presence of intact, lumps of damaged starch granules with hilum enlarged and depressed after processing; (**i**,**j**) Starch granule from GC36 (Sauveterrian); (**k**) Starch granule from GC14 (Sauveterrian); (**l**) Lump of polyhedral starch granules of Foxtail millet (*Setaria italica*); (**m**–**o**) Starch granules from GC44 (Neolithic); (**p**,**s**) Circular starch granules of modern *Triticum monococcum* showing a bimodal distribution; (**q**) Starch granule from GC44 (Neolithic); (**r**) Starch granule from the calculus deposit of GC44 (Neolithic); (**t**) Starch granule from the calculus deposit of GC44 (Neolithic).

Of 16 individuals with micro-debris in dental calculus, only 6 individuals contained identifiable starch granules (the number of identified starch granules are provided in brackets): Late Upper Palaeolithic/ Late Epigravettian individuals GC60 (n = 2) and GC33 (n = 1), Early Mesolithic/Sauveterrian individuals GC14 (n = 2), GC28 (n = 8), and GC36 (n = 93), and Early Neolithic individual GC44 (n = 35) (see Fig. 6, Table 2). Two different morphotypes of starch granules have been identified in the dental calculus of these individuals.

**Morphotype 1.** Such morphotype is represented by single large, round to sub-oval starch granules ranging between 21.1 and 33 μm in maximum dimensions (mean size of 31 μm), lenticular 3D shape with equatorial groove always visible, central hilum and visible lamellae, defined centric cross in cross polarised light, and small spherical and sub-spherical granules with a central hilum. Starch granules of the Triticeae tribe are characterised by a bimodal distribution involving the presence of large, round to sub-oval A-Type granules such as those identified in our sample and small spherical and sub-spherical granules with a central hilum (≤ 10 μm) known as B-type granules^48–50^. While the latter ones have not been identified in association with the large granules, the dimension and features of them are consistent with the Type A grains of the Triticeae tribe.

Of 16 analysed individuals, 2 individuals yielded starch granules assigned to the Triticeae tribe in their calculi (see Table 2). Most of the Type A grains in our sample appeared damaged, and this may be linked to food processing or enzymatic digestion.

**Morphotype 2.** Starch granules attributed to this morphotype have only been identified in 4 individuals (see Table 2). They are characterised by a polyhedral to sub-polyhedral 3D morphology, a central hilum with fine cracks sometimes extending across all granules and are very well known in ancient starch research (e.g.,^51,52^). Such starch granules were assigned to the Paniceae tribe, as they were smaller (never above 21 microns) and within the size range found in species of *Setaria* spp., *Panicum* spp., and *Echinochloa* spp.^53^ and also appeared more flattened. Our reference collection has species of plants of the genera *Setaria* and *Echinochloa* that grow well nearby water environments. It is very likely that a mixture of species from such genera contributed to the diet as species of the Poaceae family might have been common in the cave environs already by the end of the Palaeolithic^54^. Furthermore, they often grow in association with each other.

### Plant and animal tissues

Fragments of wood elements were found in four individuals (Table 2). Among them, this study has identified a fragment of Gymnosperm tracheid with bordered pits (softwood) in the Epigravettian individual GC21, a fragment of a burnt tracheid embedded within the calculus matrix of the Epigravettian individual GC60 and an Angiosperm vessel (hardwood) in the Neolithic individual GC27. Microcharcoal and burnt debris were found in 81% of the samples while isolated plant fibres and vegetal tissues were found in 69% of the analysed sample still embedded in dental calculus. The identification of these elements was not possible due to the absence of diagnostic features^55^. A phytolith trapeziform/pyramidal faceted short cell was found in the Epigravettian individual GC58. Bast fibres are generally absent, but it is interesting to note that non-diagnostic plant fibres are damaged, suggesting that they were processed (chewed or worked before they entered in the mouth). Spores were found in four samples (GC14, GC15, GC28, and GC44). The spores’ morphotypes are compatible with those belonging to *Alternaria* spp. and *Cladosporium* spp. (Phylum Ascomycota). These species are fungi ubiquitously found in indoor and outdoor environments and many species are plant pathogens^56^.

With regard to the animal micro remains, a fragment of a barbule (part of the feather) was found in the Late Mesolithic/Castelnovian individual GC8. Based on the morphology of the nodes (ring-shapes), this element was attributed to the Galliformes order^57^. The finding of a galliform feather fragment in the calculus of the individual GC8 is not directly reflected in the avian bone assemblage of the Castelnovian levels. However, since this taxon is documented both in the Neolithic and the Sauveterrian levels, it was very likely present in the area also during the Castelnovian period.

## Discussion

The multipronged analyses applied to the rich dental record from Grotta Continenza allowed us to identify differences among the Late Upper Palaeolithic and Mesolithic foragers and Neolithic farmers in terms of dental metric traits, pathologies, and dental wear patterns, as well as in the type fo plant foods preserved in the dental calculus.

The reduction trend in molar dental size observed at Grotta Continenza fits well with the previously observed post-Pleistocene dental reduction^6,58,59^. The gracilization of teeth is possibly linked with the population replacement in the Fucino Basin area at the end of the Castelnovian cultural horizon. At Grotta Continenza, this is also supported by aDNA results^29^. Three individuals analysed for aDNA were associated with the Mesolithic occupation at the site—two come from the Sauveterrian phase of occupation and one from the final Castelnovian phase. All three individuals show typical European Western Hunter-Gatherer (WHG) ancestry. There are five Neolithic individuals from the site that were analysed for aDNA and can be modelled as a two-way mixture of 5% of local hunter-gatherer ancestry and 95% Neolithic Anatolian ancestry with a small proportion of ancestry coming from Iranian Neolithic farmers and Caucasus hunter-gatherers. This suggests that while we should probably account with a rather limited level of admixture between the local foragers and Early Neolithic farmers at the start of the eight millennium cal BP, there must have been a significant influx of Neolithic farmer populations at this time that contributed considerably to new lifeways of people whose remains were found buried at Grotta Continenza.

Differences observed in the macrowear could be related either to less abrasive foods in the Neolithic period and/or to a different age-at-death structure of the subsamples among periods. On the basis of this evidence, we could assume a technological advancement in the Neolithic food technology involving different modalities of plant food acquisition and processing techniques, overall leading to softer and less abrasive food, contrary to what has been observed by other scholars^60,61^. However, the alternative hypothesis of different demographic profiles cannot be excluded, where Neolithic individuals might have had a shorter lifespan on average. This hypothesis could be contradicted by the suggestion of a higher caries prevalence in the Neolithic period, a condition that accumulates through ages. The higher caries prevalence in the Neolithic period could be related to an increased consumption of cariogenic foodstuffs, even through a more complex aetiology of caries can be related to hormonal influences and, more in general, to environmental and cultural stress levels^62^. However, it should be emphasised that caries and LEH data are strongly affected by the high degree of macrowear and could be biased by differential demographic profiles among the chrono-cultural horizons.

Also, the microwear pattern observed in the Grotta Continenza dental assemblage does not follow the expected trends. Contrary to the common wisdom that foresees higher meat consumption (resulting in a reduced density of striations) in the earliest chronological periods and more abrasive diets during the Neolithic^14^, at Grotta Continenza we observed a reduction in the scratch density in the Neolithic group. The scratch density remains stable from the Epigravettian to the Mesolithic periods, changing significantly during the Neolithic only.

When analysing as a whole the patterns of scratches by means of rPCA, the differences are present between the periods but are not pronouncedly marked. These differences are much more evident in the premolar teeth than in the molars. Pérez-Pérez et al.^8^ observed that similar tool technologies may cause comparable microwear patterns independently from climatic conditions and available foodstuff. They also noted that buccal microwear patterns can depend on the type of stone technology used, with fewer scratches found in more advanced technocomplexes. Other authors^11,63^ showed that buccal microwear analysis is a rather effective tool in distinguishing human groups characterized by well-differentiated subsistence economies. At Grotta Continenza, we observe a large overlap in buccal microwear patterns between different chrono-cultural horizons. Therefore, the sole buccal microwear analysis in this context is not enough to efficiently discriminate between the teeth (individuals) dated to different periods. Despite the profound cultural diversity and technological transitions observed throughout 8000 years of site occupation, at Grotta Continenza the microwear analysis suggests an overall homogeneity in the staple food availability and exploitation.

On the other hand, clear differences in the selection of edible plant foods can be identified through the application of dental calculus analysis. We observe a familiarity with the consumption of plant foods since the Epigravettian as well as differences in types of plants used between the Early Mesolithic (Sauveterrian) and Early Neolithic individuals. In particular, the micro-fossils extracted from the mineralized biofilm of 16 out of 21 sampled individuals and 6 individuals with preserved starch granules in their plaque reveal that the populations of Grotta Continenza consumed plants of the Triticeae and Paniceae tribes since the Upper Palaeolithic and throughout the Mesolithic (Sauveterrian) and Neolithic.

The consumption of seeds of the Paniceae tribe is particularly well attested in one Mesolithic individual by the recovery of more than 80 starch granules well preserved in the calculus matrix. While archaeobotanical studies have not been reported for Grotta Continenza, paleoenvironmental comparisons can be made with two pollen analyses carried out in the Fucino Basin^54^ and in Piano Locce, situated on the southern slope of the Gran Sasso^64^ as well as with current vegetation studies^65,66^ in order to document ecological changes between the end of the Pleistocene and the beginning of the Holocene until the present-day. Such studies confirm the presence of the botanical family Poaceae, without indicating their genus, since the Last Glacial Maximum in the Fucino region.

In contrast, with our starch granule analysis of dental calculus we were able to identify the Paniceae and Triticeae tribes, which are part of the Poaceae family. Compared to the current vegetation, the Paniceae tribes were probably more widespread, given the presence of the Fucino Lake, which was artificially drained in 1875. Preferring mild climates, the Paniceae tribe was presumably more widespread during the more temperate climatic phases. *Setaria* is one of the possible genera to which the starch granules recovered in the calculi can be assigned, and some species of *Setaria* (e.g. *Setaria verticillata*) grow in the Abruzzo region at altitudes up to 1000 m above sea level. The significant number of Paniceae starch granules identified in the Sauveterrian individuals from Grotta Continenza seem to indicate the use of these plants as foods. This finding corresponds to the pattern seen in other regional case studies where many of the genera of the Poaceae botanical family were used as edible plants before the introduction of domesticates (e.g.^17,47,67,68^).

No evidence related to the consumption of plant foods is preserved in the Late Mesolithic (Castelnovian) individuals. On the other hand, a strong reliance on cereals is documented in at least one individual dated to the Neolithic period. Starch granules recovered in the Neolithic sample mainly refer to Triticeae, presumably barley and wheat, the first cereals cultivated in this region. *Hordeum vulgare*, *Triticum monococcum*, and *Triticum dicoccum* are the most common cereals in the Neolithic of Abruzzo^69,70^. The cereals were probably roasted in order to achieve a better separation of the grains from the glumes. This is proven by the discovery of the charred archaeobotanical remains at various Neolithic sites in Abruzzo (e.g. Villaggio Leopardi, Catignano)^69,70^. The elimination of the glumes has certainly made the food softer and more digestible. Interestingly, many starch granules identified in the directly AMS-dated Early Neolithic individual GC44 are damaged, suggesting enzymatic digestions of the granule as well as possible processing or roasting practices of the seeds before their mechanical transformation into flour, which might have offered a softer final product included in the Neolithic diet. This would also explain the less developed level of macrowear and the reduced buccal scratch density documented in the analysed Neolithic sample. Also, a more regular intake of carbohydrates during this period could correspond to the more diffuse presence of oral pathologies such as caries, which are slightly more frequent among the examined Neolithic individuals.

A close exposure of the individuals to fireplaces (both indoor and outdoor environments) and/or the ingestion of cooked /roasted food is also indirectly suggested by the abundant presence of charcoal in almost all of the analysed calculi. Charcoal inclusions might have also contributed to the general level of abrasion documented in the buccal microwear. Wood particles, also documented in the dental calculi of all of the analysed individuals, might have been inhaled in the proximity of fireplaces or while using teeth in extramasticatory activities.

It is worth noting that the difference seen in the results of the rPCA of premolars suggests a different use of such teeth during the chewing and/or biting processes over time. This pattern could be interpreted as a change in the involvement of teeth from incisors to premolars in extramasticatory activities over time (use of teeth as third hand^71^), as testified by the extramasticatory wear and chipping scored in the anterior dentition and premolars. Indeed, wood fibres have been found in the dental calculus of individuals chronologically ranging from the Epigravettian to the Neolithic. Unfortunately, the dental assemblage from Grotta Continenza is predominantly composed of isolated teeth, thus preventing the identification of patterns of extramasticatory wear.

From the Epigravettian period to the Neolithic, the Fucino Basin experienced important changes in climate and cultural traditions of human groups occupying this micro-region, as well as in food resource availability. These changes were also accompanied by a likely population discontinuity in the transition from the Mesolithic to the Neolithic in this area ^29^. Over 8000 years that cover these developments, the environment surrounding the cave and the vicinity of the lake provided an ecological setting for different species of plant resources, the consumption of which is documented since the earliest occupation of this locale based on dental calculus and dental microwear analyses. While the consumption of species of the Poaceae family (namely, *Avena* sp.) is known since the Gravettian at the site of Paglicci (Apulia)^72^, and processing of aquatic ryzomes (i.e*., Typha latifolia*) is documented at the site of Bilancino (Tuscany)^73^ during the same period, very limited amounts of plant macro-debris were recovered in Upper Palaeolithic dental calculus from Grotta Continenza. However, the analysis carried out on this individuals from the site provided the first direct evidence of the consumption of specific grass grains of the Paniceae tribe by Early Holocene foragers in the Italian peninsula. In Sicily, dental macrowear analyses carried out on Mesolithic individuals from Grotta dell’Uzzo and Grotta della Molara^74^ confirm that plant foods were indeed consumed by Holocene foragers. However, only wild legumes (*Lathyruys* sp., *Pisum* sp.), arboreal fruits (*Arbutus unedo*), acorns (*Quercus* sp.) and wild grapes (*Vitis silvestris*) were retrieved through systematic flotation of the sediments at Grotta dell’Uzzo^75^, hence confirming the specificity of the dietary choices carried out by Early Mesolithic foragers at Continenza.

Throughout the sequence, changes in dietary practices are less visible on the basis of the faunal remains. The available raw data on the Grotta Continenza faunal assemblage^76^, reorganized according to the earlier suggested chronological attributions of various cuttings (for more details see Supplementary Text S1), do not show dramatic changes over time except for the intense focus on fishing during the Epigravettian. Even the introduction of domesticates in the Neolithic did not produce a significant modification in the diet at least based on animal species representation, since wild mammals, birds, and fish are still found in relatively high frequencies in the Neolithic levels. Hence the available faunal data are somewhat at odds with the results presented herein, which only goes to show that relying on only one strand of evidence provides a rather partial picture.

Results of this research confirm the initial hypothesis that major palaeodietary changes are identifiable among the different cultural chronostratigraphic units of Grotta Continenza. Technological changes in modes of food processing with the start of the Neolithic in the Fucino region changed dietary habits with the introduction of different processing practices, leading to softer and possibly more processed foods. Microwear and dental calculus analyses document such a shift towards less abrasive diets in the Neolithic at Grotta Continenza.

At the methodological level, our study shows how the combination of different approaches in the analysis of dental remains from Grotta Continenza allowed us to achieve different levels of resolution with regard to the identification of ancient dietary trends, which are rarely obtained by any of the applied analytical methodologies on their own. This situation strongly suggests that for a reliable and nuanced understanding of past diets based on the study of human odonto-skeletal remains we should always mobilize more than one analytical procedure, as recently put forwards by Oxilia et al. for the Late Pleistocene and Early Holocene foragers of the eastern Alpine region of Italy^77^. Different strands of analysed data sometimes tell different or even seemingly contradictory stories or accounts that provide diverse levels of detail with regard to the underlying evolutionary and cultural processes being studied. While we believe that the presented analyses of odonto-skeletal remains from Grotta Continenza provide important indications of profound changes that took place across the Pleistocene-Holocene and Mesolithic-Neolithic transitions, further exploration of this material and other strands of data from the site will continue to unravel intricacies of complex processes of human–environment interactions and major cultural transformations in this corner of the Mediterranean.

## Supplementary Information

Supplementary Information.

Supplementary Table S6.
