# Supplementary material for: Multipronged dental analyses reveal dietary differences in last foragers and first farmers at Grotta Continenza, central Italy (15,500–7000 BP)
Source: Sci Rep. 2021 Feb 19;11:4261. doi: 10.1038/s41598-021-82401-2 (PMC7895915; doi:10.1038/s41598-021-82401-2)
Supplement: Supplementary file 1 — Supplementary Information. [file 41598_2021_82401_MOESM1_ESM.pdf]

Multipronged dental analyses reveal dietary differences in last foragers and first farmers at Grotta Continenza, central Italy (15,500–7000 BP)

Alessia Nava[1,2], Elena Fiorin[1], Andrea Zupancich[1], Maria Letizia Carra[1], Claudio Ottoni[1], Gabriele di Carlo[3], Iole Vozza[3], Orlando Brugnoletti[3], Francesca Alhaique[4], Renata Grifoni Cremonesi[5], Alfredo Coppa[6], Luca Bondioli[4,7], Dušan Borić[8], and Emanuela Cristiani[1*]

[1]DANTE - Diet and ANcient TEchnology Laboratory, Department of Maxillo-Facial Sciences, Sapienza University of Rome, Rome, Italy

[2]Skeletal Biology Research Centre, School of Anthropology and Conservation, University of Kent, Canterbury, UK

[3]Department of Maxillo-Facial Sciences, Sapienza University of Rome, Rome, Italy

[4]Bioarchaeology Service, Museum of Civilizations, Rome, Italy

[5]Department of Archaeological Science, University of Pisa, Pisa, Italy

[6]Department of Environmental Biology, Sapienza University of Rome, Rome, Italy

[7]Department of Cultural Heritage, University of Padua, Padua, Italy

[8]The Italian Academy for Advanced Studies in America, Columbia University, USA

*Corresponding author. Email: emanuela.cristiani@uniroma1.it

Supplementary text S1: The Grotta Continenza site

Grotta Continenza opens onto the northern slopes of Mount Labrone, 710 m asl and 43 m above the southern limits of the present-day Fucino Basin. This region was previously occupied by a palaeolake (Lago Fucino), the level of which oscillated repeatedly during the Late Pleistocene and Early Holocene[1]. The site includes a rock-shelter about 20 m wide and 7-8 m deep, representing the main part of the cave, and an inner space, the proper cave, about 8 by 8m wide (Fig. 1). While no evidence of palaeolake shoreline (e.g. primary lake sediments) were identified inside the cave, sediments from the site have been included in the altitude belt (between 685 and 715 m asl) that documents the highest stand of the Fucino Lake[1].

Systematic excavations, carried out from 1978 to 2013 (Fig. 1), revealed a 9-metre-deep stratigraphic sequence[2-6], with early prehistoric strata documenting a continuous use of the site from the Last Glacial (ca. 15,500 cal BP) to the Early Holocene (ca. 7000 cal BP). A recent study by Boschian et al[7] provides a comprehensive overview of the stratigraphy and dating of the sequence at Grotta Continenza, suggesting the following groupings of cuttings into several main chrono-cultural units (from top to bottom): Roman period, Bronze Age, Eneolithic (cuttings 1–2), Middle and Early Neolithic (cuttings 2–22), Late Mesolithic/Castelnovian (cuttings 23–24), Early Mesolithic/Sauveterrian (cuttings 25–28), and the final phases of the late Upper Palaeolithic/Epigravettian (cuttings 29–48). This division was based on both the characteristics of the associated material culture found in each of the cuttings as well as a series of 27 radiocarbon measurements (17 conventional and 10 AMS dates) available at the time.

Since the publication of the study of Boschian et al[7], another 11 AMS dates have most recently become available for human remains analysed for ancient DNA (aDNA) ([8]: Tables S2–3). Of 11 dates, three fall in the assumed duration of the Early Mesolithic Sauveterrian phase (UCI-198583, UCI-198579, UCI-198584), one in the assumed duration of the final Mesolithic Castelnovian phase

(UCI-198574), five in the assumed duration of the Early Neolithic (UCI-198575, UCI-198580, UCI-198582, UCI-198581, UCI-213625), and two in the final phases of the Italian Eneolithic (UCI-198576, UCI-198577). While there is an overall agreement between the provenance of the human remains on which these dates are made and attributions of cuttings to chrono-cultural units suggested by Boschian et al[7], there are also several new insights provided by the new dates. They suggest that cuttings 21–23 and 14–15, assumed to have corresponded with the earliest Neolithic, contain some residual Mesolithic material of Sauveterrian and Castelnovian provenance respectively. On the other hand, there is also evidence of Eneolithic intrusions that deposited human remains dated by UCI-198577 in cutting 10. In order to alleviate somewhat possible issues with the chronological attribution of some human remains analysed in this study, especially for the remains with no information on the cutting from which they originate, we report here for the first time two new AMS radiocarbon measurements for individuals GC14 and GC44. OxA-39688 dates individual GC14 in 9351±32 BP with the calibrated range of 10,675–10,440 cal BP at 95% confidence, falling into the Early Mesolithic, Sauveterrian phase of occupation. OxA-39685 dates individual GC44 in 6837±26 BP with the obtained calibrated range of 7715–7610 cal BP at 95% confidence, falling into the Early Neolithic phase of occupation.

Over more than 8500 years, Grotta Continenza was repeatedly used as a dwelling and a funerary place. The stratigraphic sequence encompasses a total of 48 cuttings. Five hearths and abundant lithic and faunal remains indicate an intense occupation of the site with its exclusive use for daily life activities in the earlier phases of the Late Epigravettian (phases EP1 and EP2, cuttings 48–35, ca. 15,690–13,100 cal BP). Fireplaces are also documented in the later phase of the Late Epigravettian (EP3, cuttings 34–30, ca. 12,400–11,200 cal BP) when seven individuals were found buried in cuttings 34–33 and 32–31, indicating the initial use of the site for funerary practices[2,9].

During the Early Mesolithic, Sauveterrian phases (ca. 11,200–10,500 cal BP), the outer rock-shelter as well as at the entrance to the inner cave continued to be used as a dwelling place but also contained a number of disarticulated human remains. The recovery of numerous disarticulated human remains in the layers, as well as at least nine disarticulated individuals and one articulated inhumation burial in the Late Mesolithic, Castelnovian layers 23–24 (ca. 8500–7800 cal BP), indicate that the site was used for funerary practices throughout the Mesolithic.

In the Neolithic (layers 2–22, ca. 7600–6900 cal BP) dwelling activities were limited to the outer space while the inner part of the cave was destined for funerary practices only. In particular, disarticulated remains of at least 45 individuals were recovered, including many infants. No burial pits were identified during the excavations, hence corpses might have been deposed directly on the ground, without specific arrangements. The only exception is represented by an old woman whose corpse was associated with two vessels containing the incinerated remains of a 4- and 8-year-old individuals[2].

Supplementary text S2: The faunal remains at Grotta Continenza

The animal remains from Grotta Continenza have only partially and preliminarily been studied[10,11], but may provide a valuable starting point for discussion. In the present description the original data by Wilkens[10], have been grouped following the chrono-stratigraphic attributions suggested by[7]. The analyzed faunal samples belonging to the different periods are not comparable in size, although the total number of identifiable remains is never negligible (NISP=8044 for the Epigravettian; NISP=4846 for the Sauveterrian; NISP=920 for the Castelnovian; NISP=940 for the Neolithic); the 95 specimens from the uppermost cuts are not discussed here (Supplementary Table S6).

Fishing appears to have been very important, especially during the Epigravettian (Supplementary Table S6, Supplementary Fig. S9) when fish remains, in particular trout which is the only species

surely recovered at the site, account for almost 90% of the identified assemblage. In other periods, the frequency of this taxon, although still with the highest representation in each sample is "only" between almost 40% in the Mesolithic and 33% in the Neolithic.

Edible mollusks (in particular *Helix ligata*) show a relatively high percentage only in the Mesolithic; marine taxa are present in low frequencies, only used as ornaments and therefore did not represent a food source.

Birds are apparently not very significant during the Epigravettian, but such a low percentage may have been "flattened" by the extremely high frequency of fish; in later other periods the frequencies of birds vary between almost 20% in the Mesolithic and 12.3% in the Neolithic. However, more detailed taphonomic analysis are needed to confirm the actual use by humans of all the avian resources, because some taxa, especially raptors, may have nested in or around the cave, while others may have been the result of predations by other birds or carnivores. According to[10], only a mallard's element displays filleting marks. Differences among the periods in bird taxa representation may reflect shifts in human hunting activities and/or intensity of the cave's use, as well as climatic variations through time: Anseriformes, Galliformes and Passeriformes are prevalent in the Epigravettian; Passeriformes, Anseriformes, and Gruiformes in the Mesolithic; Gruiformes, Columbifomes, and Passeriformes in the Neolithic.

Except for the likely intrusive small rodents, probably mainly resulting from pellets or burrowing animals, which are almost always present in relatively high percentages, the small mammals (e.g. hedgehog, squirrel, dormouse, porcupine, hare) and carnivores (e.g. wild cat, fox, wolf, badger, marten, otter, bear) whose exploitation by humans have been documented at other sites (e.g. Arene Candide[12]; Romanelli[13,14]; Grotta Maritza[15,16]), were apparently never very important throughout the archaeological sequence; furthermore, in this case too without a detailed taphonomic study, it is not possible to assess the agent responsible for the accumulation of small mammal bones in the cave since

at other contemporaneous sites in the Fucino Basin, such as Grotta di Ortucchio and Grotta la Punta, the smaller taxa were manly accumulated by predators[17,18] . The dog is relatively frequent in the Neolithic. However, it is worth mentioning the presence of cutmarks on bones of this species in particular on some skeletal elements of a 20-24-month-old individual recovered in cuts 18-21. The presence of this animal as well as of other skeletons of young ovicaprines and pigs all recovered in specific areas close to the cave walls may be connected to the funerary use of the site during this period[10,19].

As far as the ungulates are concerned, cervids, especially red deer represented the main taxon in the Epigravettian and in the Mesolithic followed by suids. Only in the Neolithic, ovicaprines became prevalent, again followed by suids that in this case include both wild boar and pig, with the latter being more common with a ratio of about 1:6. However, cervids are still relatively abundantly documented in the Neolithic, along with fish and birds. Hence, the exploitation of wild resources still continued in this period. Both aurochs and cattle by each period are very rare, possibly in relation to the surrounding environment that was probably less suitable for large bovids. The final Pleistocene colder climatic conditions referable to the Epigravettian are documented by the presence of ibex and chamois in slightly higher numbers than in later periods.

Seasonality based on trout remains[10], indicates that the capture of this fish occurred mainly in spring and summer during the Neolithic, in spring and winter in the Mesolithic, while there is a prevalence of winter fishing activities in the Epigravettian (Supplementary Fig. S10). Autumn captures are always underrepresented throughout the archaeological sequence. Nevertheless, although samples size of "readable" vertebrae varies greatly by period, and therefore this information needs to be taken with caution, there may be an indication of a shift in the season of fishing activities from one period to the other. However, such seasonal data do not necessarily correspond to only one season of the site's occupation, because, as indicated by archaeozoological studies in other contemporaneous sites

in the Fucino Basin[15,20], at least during the Palaeolithic and Mesolithic, people were exploiting different types of resources (ungulates, birds, fish) according to the season, remaining in the Fucino area all year round and possibly only moving from one site to the other, in contrast to Barker's hypothesis of long distance movements[21,22], but in agreement with Radmilli's idea[23,24] that the influence of the lake on the local climate allowed adequate living conditions all year round.

As mentioned before, detailed taphonomic analyses are unfortunately still lacking for the Continenza animal bone assemblage; these would have been useful to better evaluate the actual species used as food as well as the intensity of site occupation in different periods. In fact, the study of other faunal samples from the same area suggest that the supposed change in human diet at the end of the Upper Palaeolithic with a marked increase of small mammals, especially hare, hedgehog and marmot (cf.[25]), did not actually occur since most of these taxa had been accumulated by carnivores, including small ones[17,18]. Furthermore, during the Upper Paleolithic and Mesolithic, the caves around the lake, analyzed in more detail from a zooarchaeological and taphonomic point of view (see[15,17,20,26]; for a general considerations and discussion about site function and seasonality see also[27]), showed differences in intensity of occupation, possibly corresponding to a different type of use, both among them and in the course of the stratigraphic sequences.

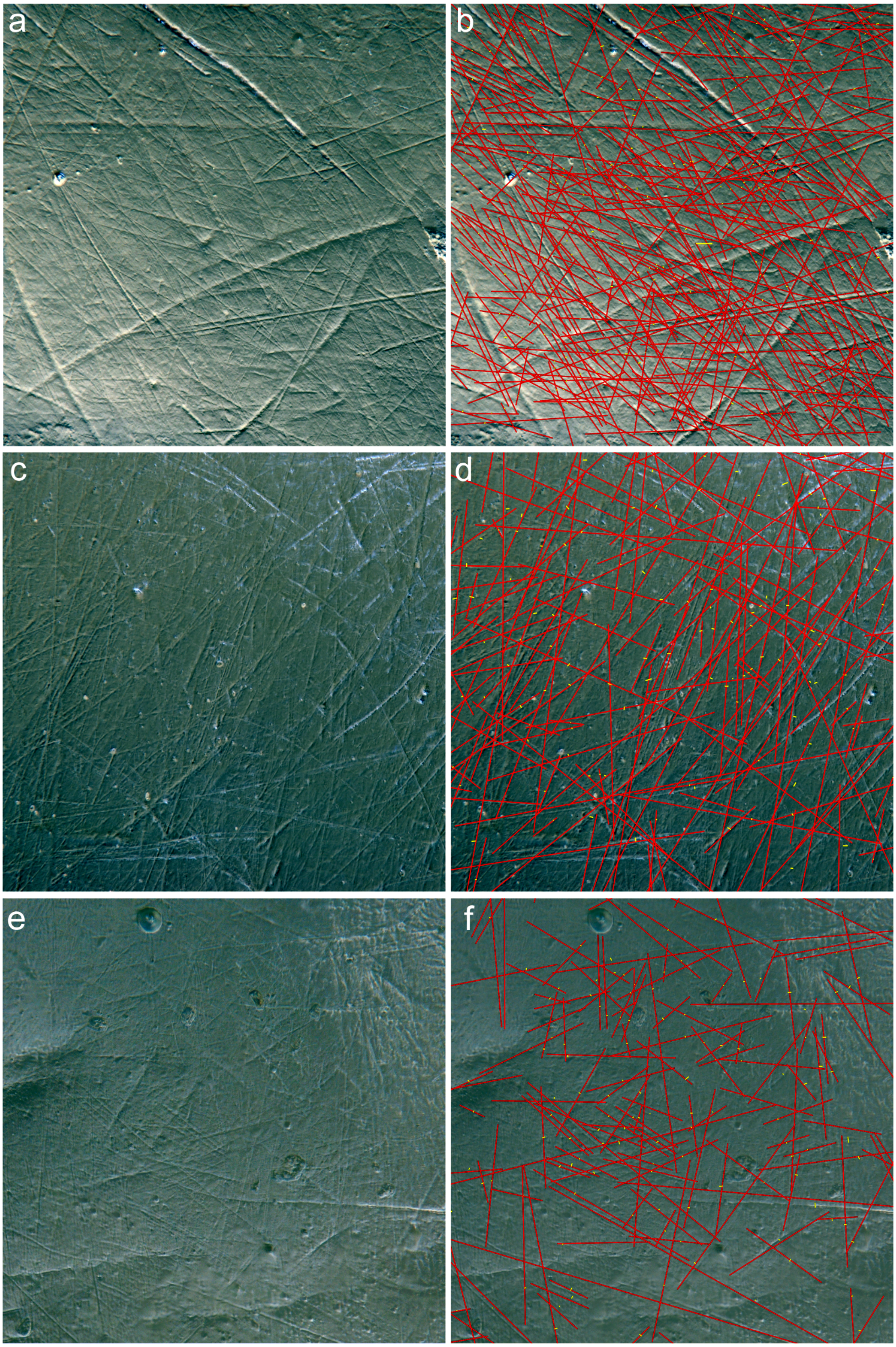

Supplementary Fig. S1: MicroWeaR Delphi examples of scratches tracing. a, b) GC 61G, EP, lower fourth permanent premolar, density = 344; c, d) GC 76A, MES, upper first permanent molar, density

= 160; e, f) GC 12A, NEO, upper fourth permanent premolar, density = 133. Region of Interest of all images 748x748 µm.

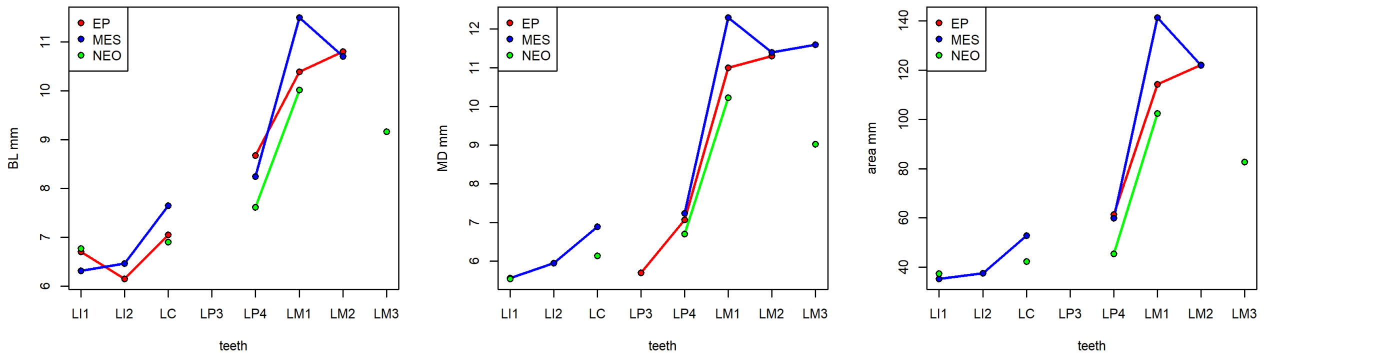

Supplementary Fig. S2: variation of the crown diameters and area of the lower dentition through the periods. EP=Late Epigravettian, MES=Mesolithic (Sauveterrian and Castelnovian); NEO=Early and Middle Neolithic.

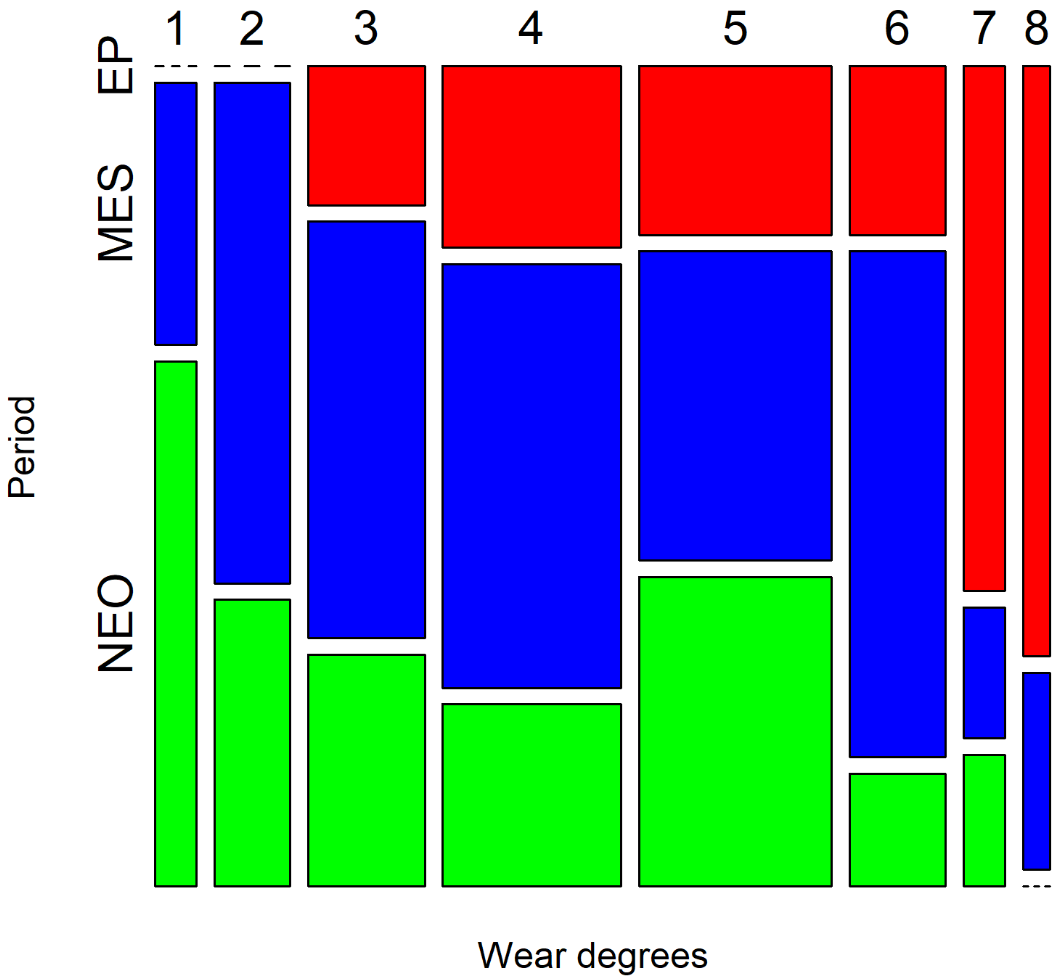

Supplementary Fig. S3: Mosaic plot of the wear degrees (for all tooth classes) by period. EP=Late

Epigravettian (n=24), MES=Mesolithic (Sauveterrian and Castelnovian, n=45); NEO=Early and Middle Neolithic (n=33).

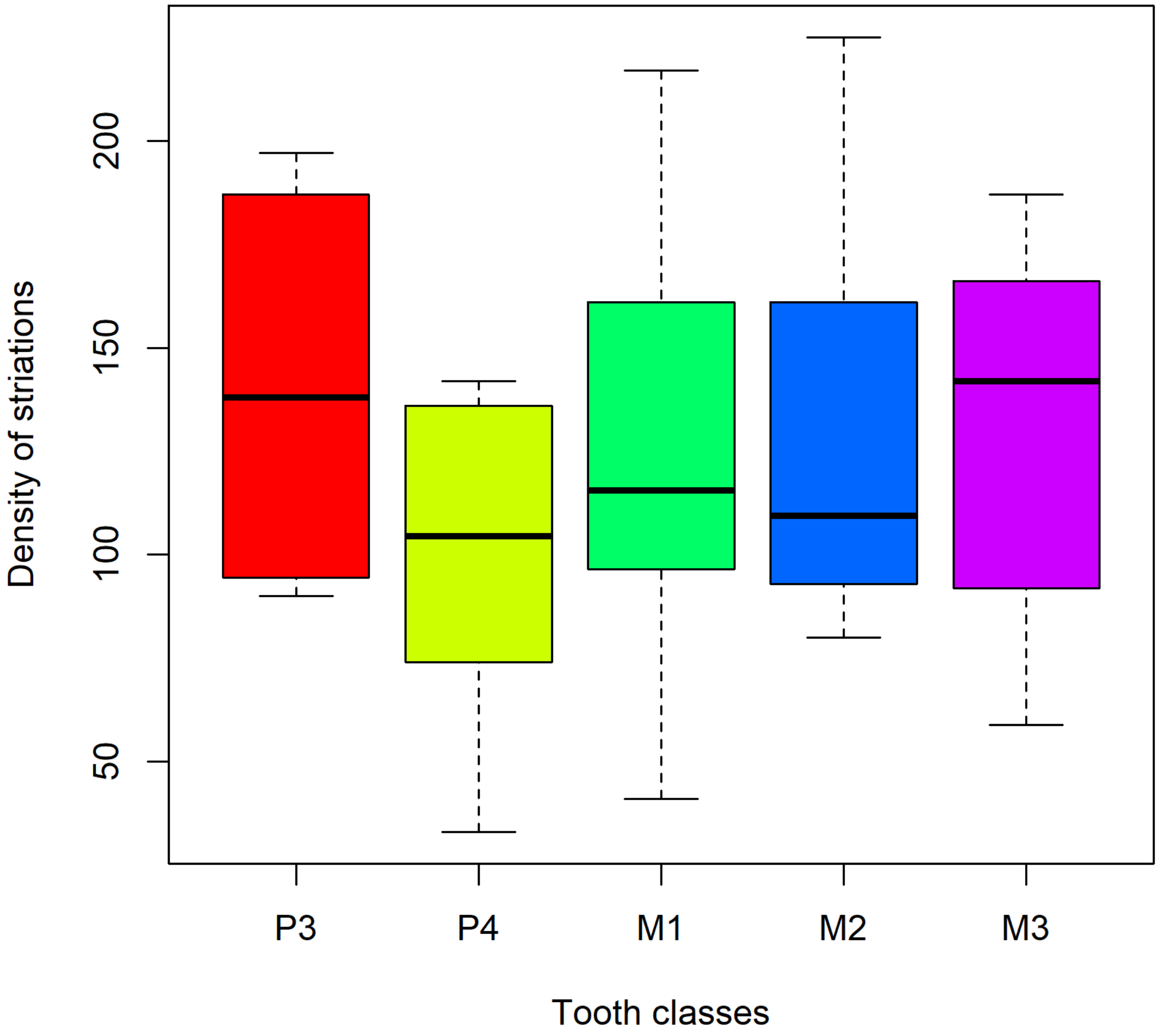

Supplementary Fig. S4: Density of scratches by tooth classes.

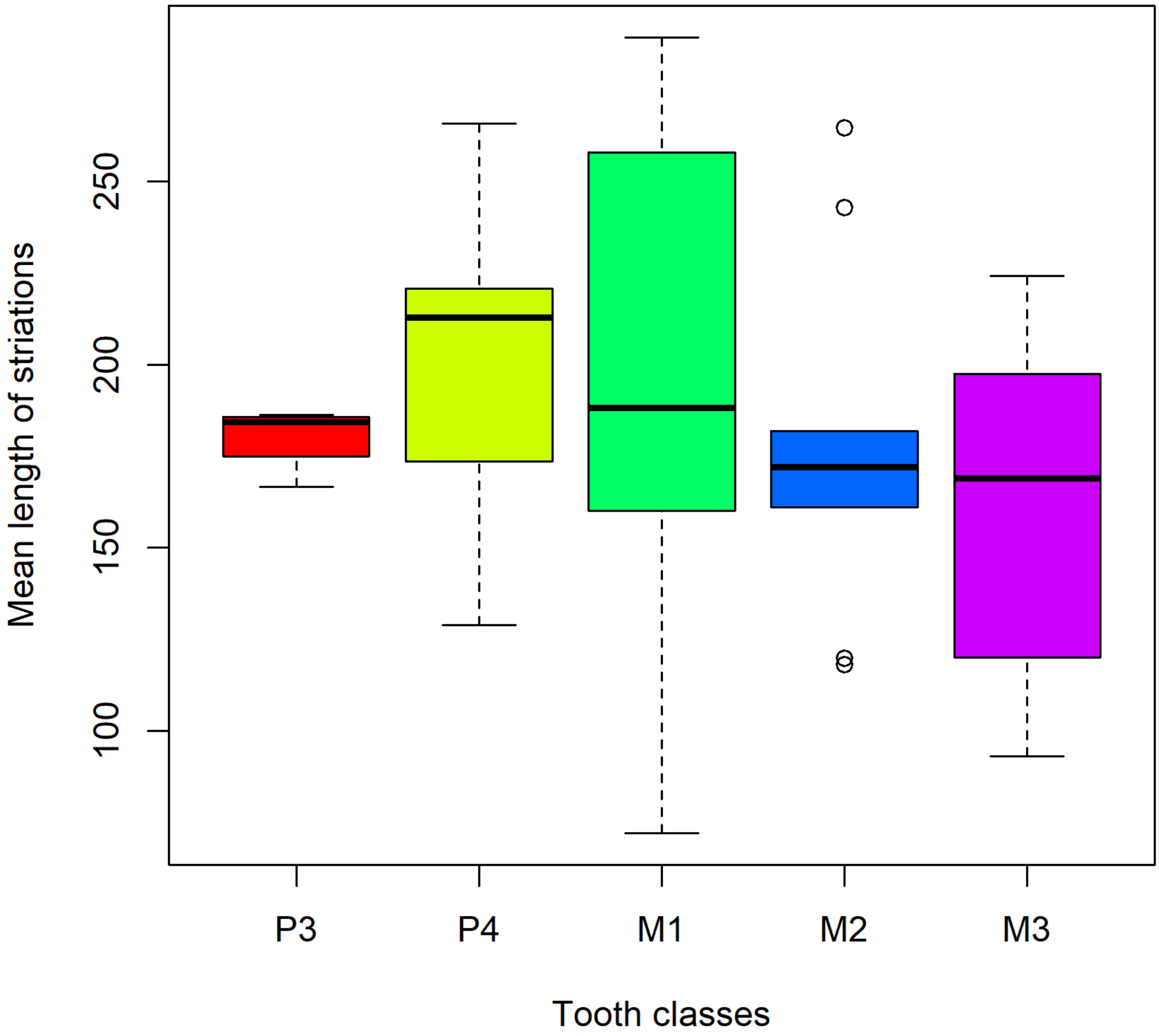

Supplementary Fig. S5: Mean length of scratches by tooth classes.

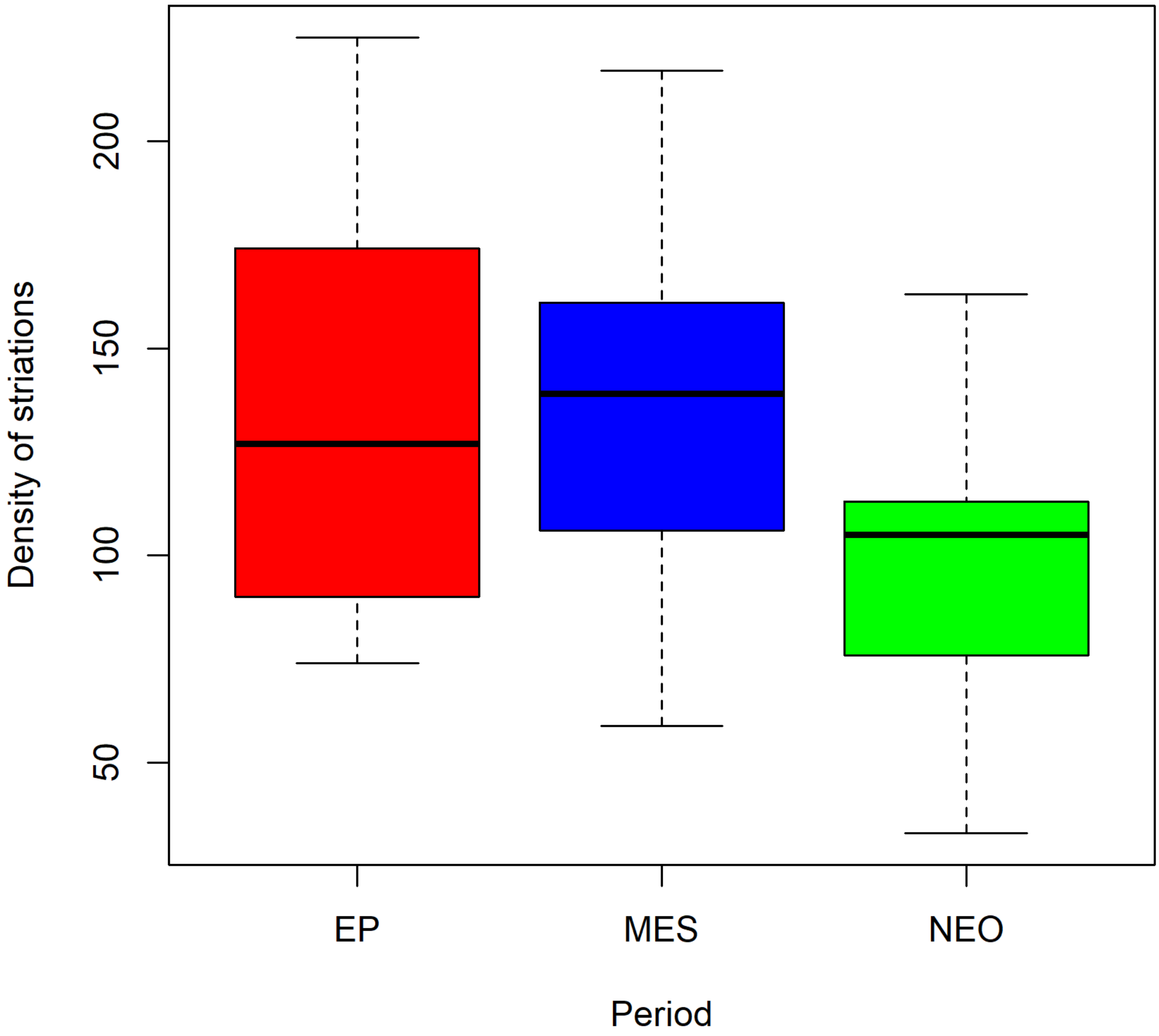

Supplementary Fig. S6: Density of scratches by period.

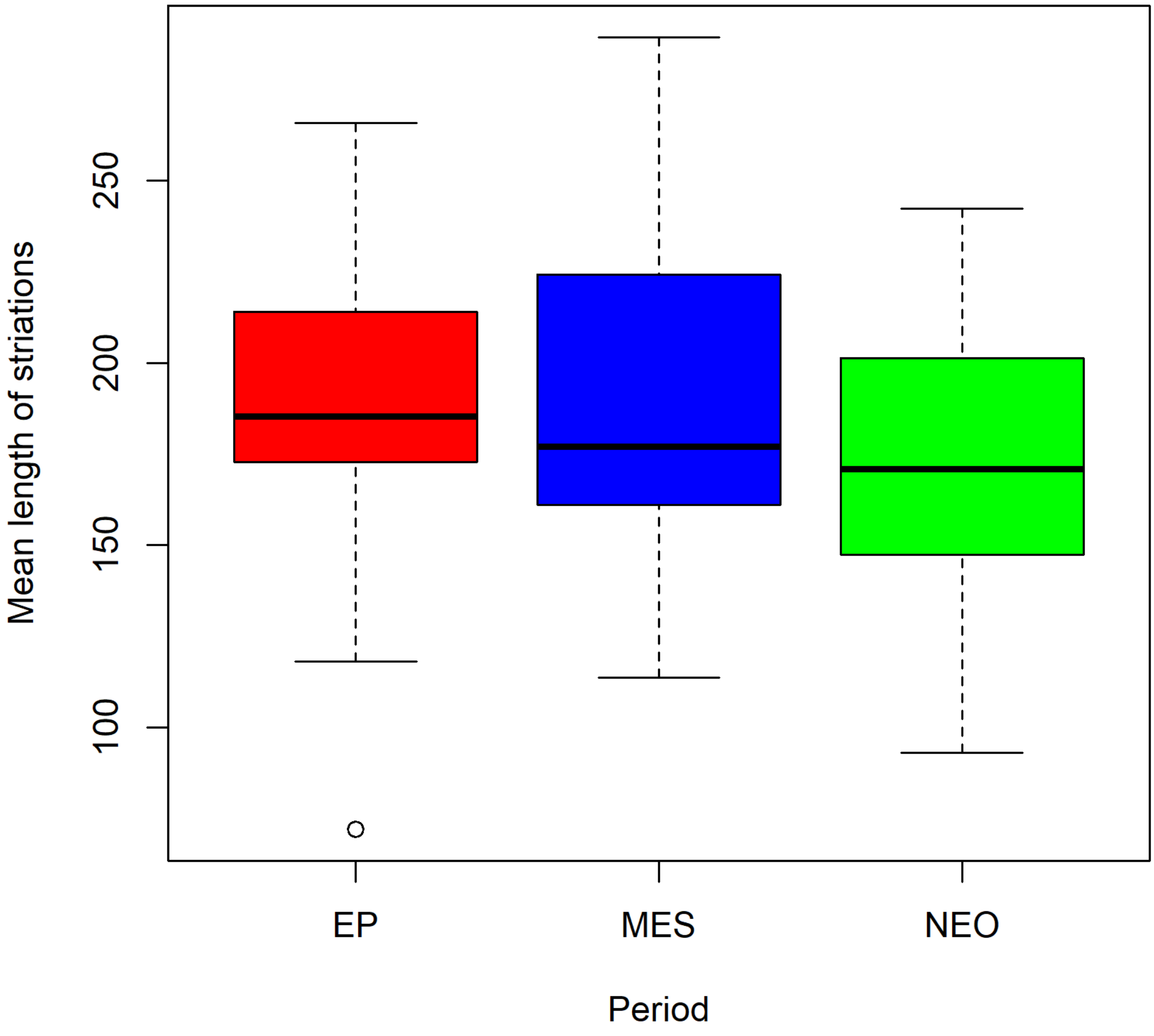

Supplementary Fig. S7: Mean length of scratches by periods.

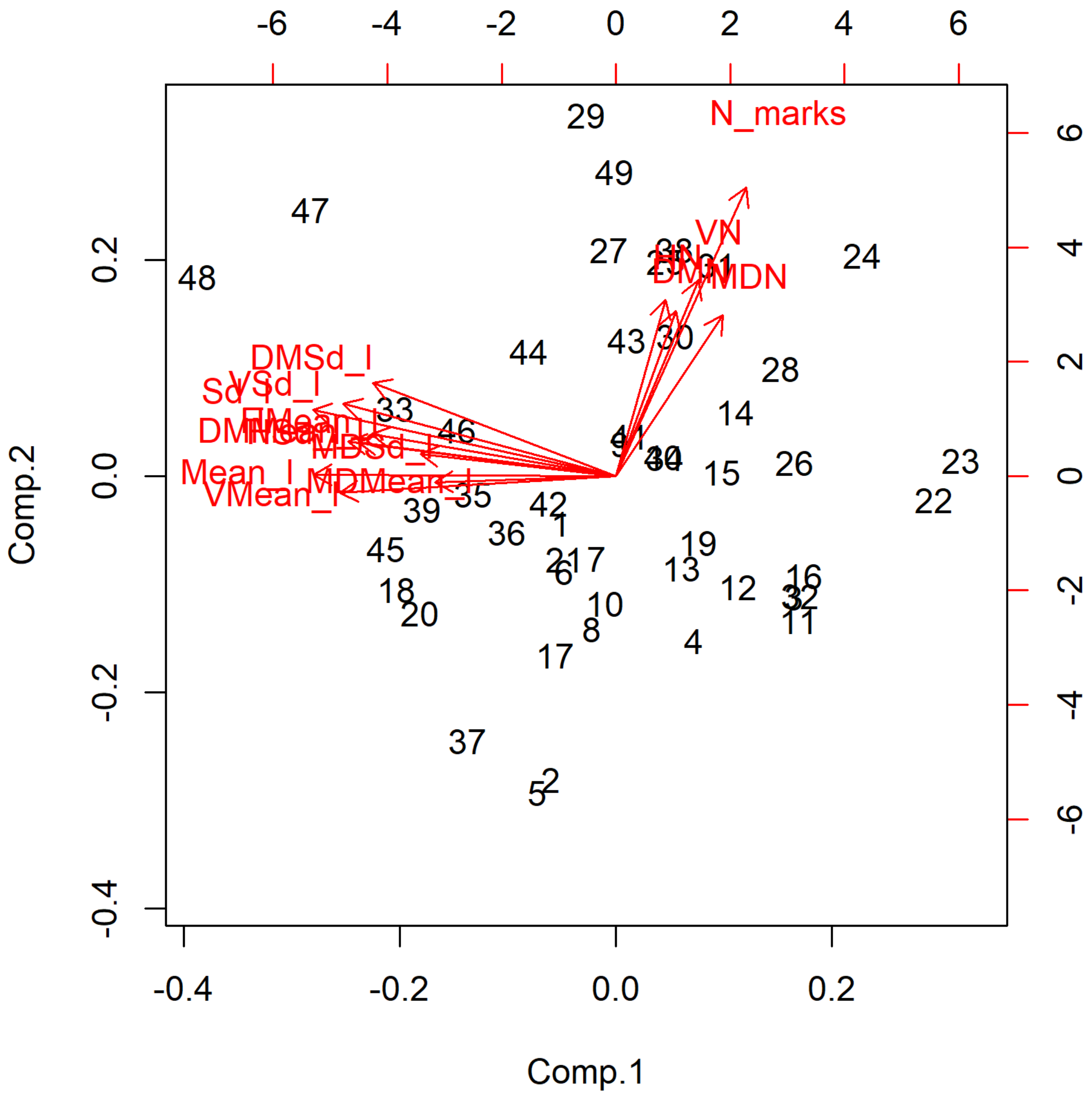

Supplementary Fig. S8: Scatterplot representing both the observations and variables of the robust Principal Component Analysis of the complete data set of buccal scratches.

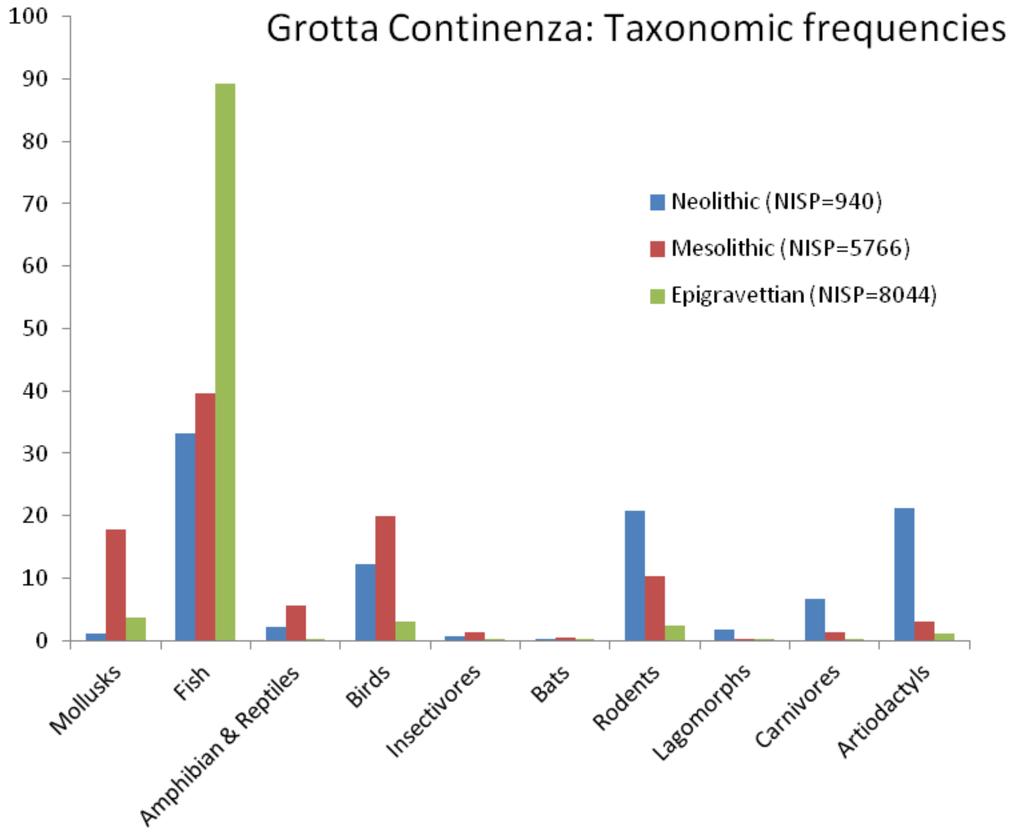

Supplementary Fig. S9: Taxonomic frequencies by Number of Identified Specimens (NISP) in the different periods.

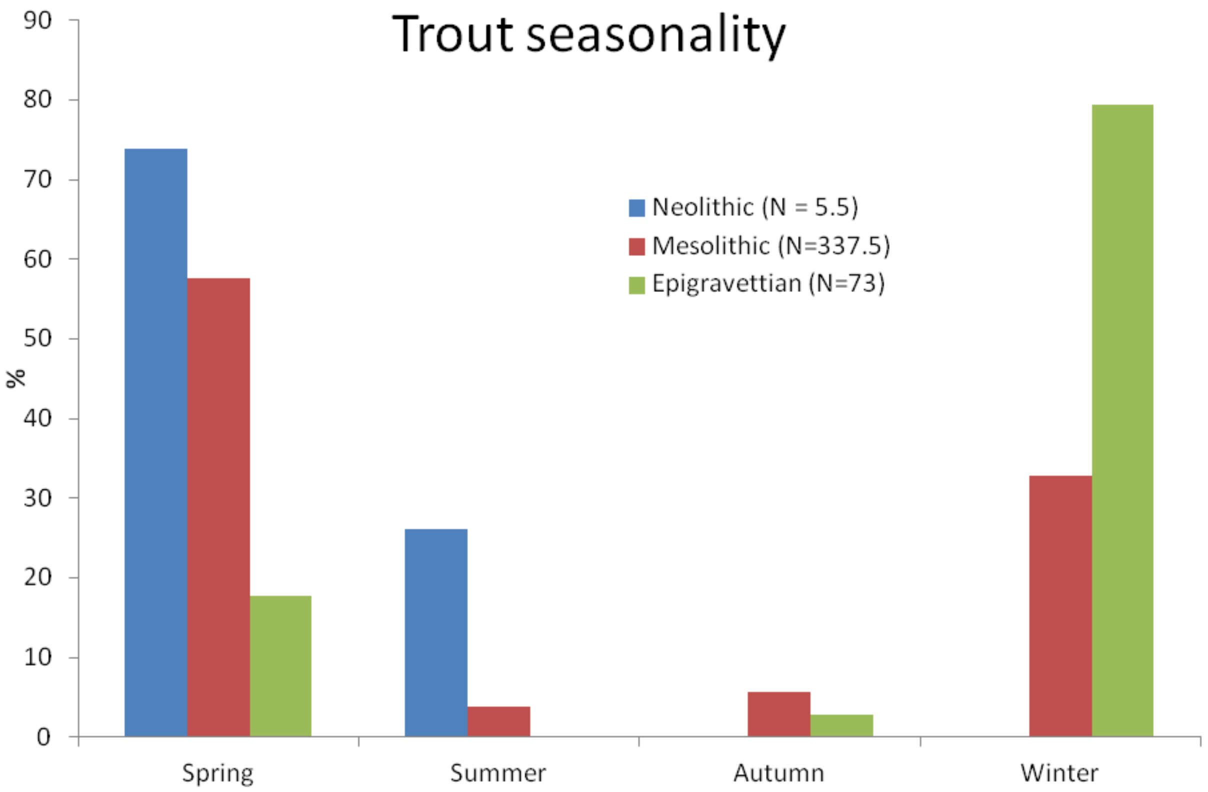

Supplementary Fig. S10: Seasonality data based on trout vertebrae in different periods (n= Number of specimens; the decimals indicate that some specimens were attributed to more than one cutting and therefore the value has been divided accordingly).

Supplementary Table S1: The dental sample by tooth types.

**Tooth type**	**n**
**UI1** (Upper Central Incisor)	**13**
**UI2** (Upper Lateral Incisor)	**7**
**UC** (Upper Canine)	**6**
**UP3** (Upper Third Premolar)	**7**
**UP4** (Upper Fourth Premolar)	**8**

**UM1** (Upper First Molar)	**9**
**UM2** (Upper Second Molar)	**9**
**UM3** (Upper Third Molar)	**8**
**LI1** (Lower Central Incisor)	**7**
**LI2** (Lower Lateral Incisor)	**5**
**LC** (Lower Canine)	**11**
**LP3** (Lower Third Premolar)	**4**
**LP4** (Lower Fourth Premolar)	**10**
**LM1** (Lower First Molar)	**9**
**LM2** (Lower Second Molar)	**11**
**LM3** (Lower Third Molar)	**4**
**Udi2** (Upper deciduous lateral incisor)	**2**
**Udm1** (Upper deciduous first molar)	**1**
**Udm2** (Upper deciduous second molar)	**1**
**Ldm1** (Lower deciduous first molar)	**1**
**Ldm2** (Lower deciduous second molar)	**1**
**M1/M2** (heavily worn and fragmented first /second, lower /upper molar)	**2**
**I1/I2** (heavily worn and fragmented central/lateral, lower /upper incisor)	**1**
**Undetermined fragment of a dental crown**	**2**
**Supernumerary peg shaped anterior tooth**	**1**
**Total**	**140**

Supplementary Table S2: Grotta Continenza archaeological labels and chronological periods.

Label	Archaeological label	Cutting	Chronological periods	Tooth type
GC 1 A	Grotta Continenza -2 -6			LRP3
GC 1 B	Grotta Continenza -2 -6			LRP4
GC 2	Grotta Continenza A 3-4	24	CAST	LLM1
GC 3	Grotta Continenza -3A			LLI1
GC 4	Grotta Continenza			URI2
GC 5	Grotta Continenza			LLM2
GC 6	Grotta Continenza		SAUV	LRC
GC 7	Grotta Continenza		SAUV	ULM3
GC 8 A	CT ripulitura A 3-4	24	CAST	ULM2
GC 9 A	Grotta Continenza rimosso	2	NEO	ULP3
GC 9 B	Grotta Continenza rimosso H 7-8	2	NEO	LRC
GC 9 C	Grotta Continenza rimosso H 1	2	NEO	LRM3
GC 9 D	Grotta Continenza rimosso H 1 rip	2	NEO	LRC
GC 9 E	Grotta Continenza rimosso	2	NEO	ULI2
GC 10 A	Continenza rimosso A-2	30	EP3	URC
GC 10 B	Continenza rimosso A-2	30	EP3	URP3
GC 10 C	Continenza rimosso A-2	30	EP3	URP4
GC 10 D	Continenza rimosso A-2	30	EP3	URM1
GC 10 E	Continenza rimosso A-2	30	EP3	URM2
GC 11 B	Grotta Continenza rimosso H 1 rip		NEO	LRP4
GC 11 C	Grotta Continenza rimosso H 1 rip		NEO	LRM1
GC 11 D	Grotta Continenza rimosso H 1 rip		NEO	LRM2
GC 11 E	Grotta Continenza rimosso H 1 rip		NEO	LRM3
GC 12 A	Grotta Continenza rimosso H 1 rip		NEO	ULP4
GC 12 B	Grotta Continenza rimosso H 1 rip		NEO	ULM1
GC 12 C	Grotta Continenza rimosso H 1 rip		NEO	ULM2
GC 12 D	Grotta Continenza rimosso H 1 rip		NEO	ULM3
GC 13 A	Grotta Continenza		NEO	URC
GC 13 B	Grotta Continenza		NEO	URP3
GC 13 C	Grotta Continenza		NEO	URP4
GC 13 H	Grotta Continenza		NEO	LRP4
GC 14 A	Grotta Continenza BB-DD		MES	URI1
GC 14 B	Grotta Continenza BB-DD		MES	URI2
GC 14 C	Grotta Continenza BB-DD		MES	URC
GC 14 D	Grotta Continenza BB-DD		MES	URP3
GC 14 E	Grotta Continenza BB-DD		MES	URP4
GC 14 F	Grotta Continenza BB-DD		MES	URM1
GC 14 G	Grotta Continenza BB-DD		MES	URM2
GC 14 H	Grotta Continenza BB-DD		MES	URM3

GC 15 A	CT	14-15	NEO	URM1
GC 15 B	CT	14-15	NEO	URM3
GC 16 A	G. Continenza -4,40		EP	LLP3
GC 16 B	G. Continenza -4,40		EP	LLM2
GC 17 A	G. Continenza BC -2 -1 0		EP	LRM1
GC 18	G. Continenza ED 1-2	35	EP	LRM2
GC 19	CT -2C	31	EP	URP4
GC 20	CT -2C	31	EP	URM2
GC 21	CT	30	EP	LRM1
GC 22	G. Continenza C/-2	31	EP	URP3/P4
GC 23 A	CT B 7	24	CAST	ULC
GC 23 B	CT B 7	24	CAST	LLC
GC 23 C	CT B 7	24	CAST	URM3
GC 24	Grotta Continenza -1A	42	EP1	LRP3
GC 25	Grotta Continenza ρ 4 x	25-27	SAUV	LRM1
GC 26 A	CT A 6	24	CAST	URI2
GC 26 B	CT A 6	24	CAST	LRM3
GC 27 A	CT GH 7/8	4	NEO	ULI2
GC 27 B	CT GH 7/8	4	NEO	ULP3
GC 27 C	CT GH 7/8	4	NEO	ULP4
GC 27 D	CT GH 7/8	4	NEO	ULM1
GC 28 A	CT CD 1-0	25	SAUV	ULM1
GC 28 B	CT CD 1-0	25	SAUV	ULM2
GC 28 C	CT CD 1-0	25	SAUV	ULM3
GC 29	Continenza B 5	24	CAST	LRC
GC 30	CT B B	24	CAST	LRM2
GC 31 A	CT -1α	28	SAUV	LLM1
GC 31 B	CT -1α	28	SAUV	LLM2
GC 33	Continenza CD -1-2	36	EP2	LLM2
GC 34	CT A 4	24	CAST	LLP4
GC 35 A	Grotta Continenza	9	NEO	ULI2
GC 35 C	Grotta Continenza	13	NEO	fragment molar
GC 36 A	Gt. CT AB -1	25-26	SAUV	molar
GC 36 B	Gt. CT AB -1	25-26	SAUV	molar
GC 36 C	Gt. CT AB -1	25-26	SAUV	LLM1
GC 37	CT	23-25	SAUV	ULP4
GC 38	CT BA 6	23	CAST	LLI1
GC 39 A	CT D 10	5	NEO	LRI1
GC 39 B	CT D 10	5	NEO	LRI2
GC 39 C	CT D 10	5	NEO	LRC
GC 39 D	CT D 10	5	NEO	LRP4
GC 39 E	CT D 10	5	NEO	ULI1
GC 40	Continenza A-1	28-29	SAUV	LRP4
GC 41 E	CT	24-25	CAST_SAUV	LRM1

GC 42	CT 3GG	2	POST NEO	molar
GC 43	CT A 6	27	SAUV	LLM2
GC 44 A	CT EF 9-10	5	NEO	LLC
GC 44 D	CT EF 9-10	5	NEO	LLM1
GC 44 E	CT EF 9-10	5	NEO	LLM2
GC 44 F	CT D 2-3-4	4	NEO	ULI1
GC 44 N	CT D 2-3-4	4	NEO	ULM1
GC 45	Continenza A 6	26	SAUV	URI1
GC 46	CT DE 10	2	NEO	URI1
GC 47	CT α 6	26	SAUV	ULP3
GC 48 A	C 6	24	CAST	LLI1
GC 48 B	C 6	24	CAST	ULI1
GC 49	Continenza A 3	23	CAST	LLI2
GC 50	Continenza B 5-6	23	CAST	URM2
GC 51	C 4-5	23-24	CAST	URI1
GC 52	D 9-10	7-9	NEO	LLI1
GC 53	Continenza D 4	24	CAST	ULI1
GC 55	Continenza A 6	23	CAST	URM3
GC 56	D 9-10	23	CAST	URP4
GC 57	Grotta Continenza	28-29?	SAUV	ULI1
GC 58	CT A/-1	29-30	SAUV-EP3	LLP4
GC 59	GT -1/B	29-30	SAUV-EP3	LLC
GC 60	CT 2A α-3	30	EP3	URM3
GC 61 C	GC γ-1	30	EP3	LRI1
GC 61 E	GC γ-1	30	EP3	LRC
GC 61 G	GC γ-1	30	EP3	LRP4
GC 61 I	GC γ-1	30	EP3	LRM2
GC 61 L	GC γ-1	30	EP3	LRM3
GC 62	Gr. Continenza β-3	32	EP3	LLP3
GC 63	Continenza β/-2	32b	EP3	LRP4
GC 64	Continenza β/-2	32b	EP3	LLP4
GC 65	CT -3BB	36	EP2	LRM2
GC 66	CT -4CC	37	EP2	LLC
GC 67	CT -1A Fs	38	EP2	LLI2
GC 68	CT A8-A7	24	CAST	LLI2
GC 69	CT A8-A7	24	CAST	URI2
GC 70	CT A8-A7	24	CAST	LLC
GC 71	CT A8-A7	24	CAST	URM2
GC 72	CT BC 7-8-9	10	NEO	LLI1
GC 73	CT B 1-0	24	CAST	ULI1
GC 74	CT 3-4	24	CAST	LLC
GC 75	CT	24+(SN?)	CAST_SAUV	molar
GC 76A	CT CP 1-4	25	SAUV	URM1
GC 77	CT CD 10	7-9	NEO	URI1

GC 78	CT CD 10	7-9	NEO	ULI1
GC 79	AB 7-8-9	16-17	NEO	URI1
GC 80	Continenza A 3-4	25	SAUV	ULM1
GC 81	Continenza A 3-4	25	SAUV	ULM2
GC 82C	CT 2-5	26	SAUV	URC
GC 82D	CT 2-5	26	SAUV	URP3
GC 82E	CT 2-5	26	SAUV	URP4
GC 82F	CT 2-5	26	SAUV	URM1
GC 82G	CT 2-5	26	SAUV	URM2
GC 83B	CT 2-5	26	SAUV	LLC
GC 83D	CT 2-5	26	SAUV	LLP4
GC 83E	CT 2-5	26	SAUV	LLM1
GC 83F	CT 2-5	26	SAUV	LLM2

Supplementary Table S3: list of the variables calculated by the Delphi implementation for MicroWeaR.

Variable	Description (all measurements in µm)
**NT**	Density of scratches (number of scratches in the ROI)
**XT**	Mean length of scratches
**ST**	Standard deviation of scratches' length
**NV**	Number of vertical[1] scratches
**XV**	Mean of the length of vertical[1] scratches
**SV**	Standard deviation of the length of vertical[1] scratches
**NH**	Number of horizontal[2] scratches
**XH**	Mean length of horizontal[2] scratches
**SH**	Standard deviation of the length of horizontal[2] scratches
**NMD**	Number of oblique (mesio-distally oriented)[3] scratches
**XMD**	Mean length of oblique (mesio-distally oriented)[3] scratches
**SMD**	Standard deviation of the length of oblique (mesio-distally oriented)[3] scratches
**NDM**	Number of oblique (disto-mesially oriented)[4] scratches
**XDM**	Mean length of oblique (disto-mesially oriented)[4] scratches
**SDM**	Standard deviation of the length of oblique (disto-mesially oriented)[4] scratches

[1]angle with the occlusal plane in the range 90°± 22.5°; [2]angle with the occlusal plane in the range 0°± 22.5°; [3]angle with the occlusal plane in the range 135°± 22.5° (left lower and right upper teeth) or 45°± 22.5° (right lower and left upper teeth); [4]angle with the occlusal plane in the range 45°± 22.5° (left lower and right upper teeth) or 135°± 22.5° (right lower and left upper teeth).

Supplementary Table S4: Individuals sampled for dental calculus with their cultural attribution and the weight of the removed dental calculus (for the full details of these specimens see S1 Table).

No.	Label	Cutting (direct AMS date)	Chrono-cultural attribution	Calculus weight (mg)
1	GC9	2	Early/Middle Neolithic	2.2
2	GC27	4	Early/Middle Neolithic	2.1
3	GC44	5 (OxA-39685)	Early Neolithic	0.6
4	GC72	10	Early/Middle Neolithic	0.6
5	GC15	14–15	Early/Middle Neolithic	4.1
6	GC79	14–17	Early/Middle Neolithic	1.5
7	GC2	24	Late Mesolithic (Castelnovian)	2.3
8	GC8	24	Late Mesolithic (Castelnovian)	3.7
9	GC30	24	Late Mesolithic (Castelnovian)	1.6
10	GC71	24	Late Mesolithic (Castelnovian)	1.6
11	GC76	25	Early Mesolithic (Sauveterrian)	1
12	GC28	25	Early Mesolithic (Sauveterrian)	0.6
13	GC36	25–26	Early Mesolithic (Sauveterrian)	2.8
14	GC14 GH	no cutting (OxA-39688)	Early Mesolithic (Sauveterrian)	1.6
15	GC58	29–30	Late Epigravettian	0.7
16	GC10	30	Late Epigravettian	4.4
17	GC 21	30	Late Epigravettian	1.3
18	GC 61	30	Late Epigravettian	6.7
19	GC33	36	Late Epigravettian	1
20	GC65	36	Late Epigravettian	3.5
21	GC60	no cutting	Late Epigravettian	5.2

Supplementary Table S5: Base statistical parameters of the BL and MD diameters. EP=Late Epigravettian; Mes=Mesolithic (Sauveterian and Castelnovian); Neo=Early and Middle Neolithic; MD= Mesio-distal diamenter; BL0 = bucco-lingual diameter.

	EP BL			EP MD			EP area			MES BL			MES MD			MES area			NEO BL			NEO MD			Neo area		
	mean	sd	N	mean	sd	N	mean	sd	N	mean	sd	N	mean	sd	N	mean	sd	N	mean	sd	N	mean	sd	N	mean	sd	N
UI1	-	-	-	-	-	-	-	-	-	7,3	0,19	3	8,8	-	1	62,6	-	1	7,1	0,2	3	60,9	0,3	3	60,9	1,10	3

UI2	-	-	-	-	-	-	-	-	-	6,4	-	1	6,9	-	1	44,2	-	1	6,5	0,2	2	43,6	0,3	2	43,6	3,35	2
UC	8,4	-	1	8,0	-	1	67,5	-	1	8,3	0,74	3	7,3	-	1	55,5	-	1	8,2	-	1	61,1	0,0	2	61,1	-	1
UP3	9,8	-	1	6,4	-	1	62,7	-	1	8,7	0,25	2	6,0	-	1	51,2	-	1	9,2	0,7	2	56,1	0,5	2	56,1	8,51	2
UP4	8,9	-	1	6,7	-	1	59,7	-	1	9,4	0,37	2	6,7	-	1	64,2	-	1	9,2	-	1	65,3	-	1	65,3	-	1
UM1	11,9	-	1	11,9	-	1	141,5	-	1	12,3	0,25	2	10,5	1,29	2	129,2	13,34	2	11,1	0,7	4	114,2	0,7	4	114,2	14,68	4
UM2	11,7	0,5	2	10,1	0,99	2	118,5	16,59	2	12,2	1,08	4	9,8	0,23	4	118,9	11,87	4	10,0	-	1	86,9	-	1	86,9	-	1
UM3	12,4	-	1	9,0	-	1	111,5	-	1	11,0	0,71	4	8,0	0,59	4	88,5	9,72	4	9,4	0,1	2	79,7	0,0	2	79,7	0,34	2
LI1	6,7	-	1	-	-	-	-	-	-	6,3	0,05	2	5,6	-	1	35,3	-	1	6,8	0,3	2	37,5	0,6	2	37,5	2,31	2
LI2	6,2	-	1	-	-	-	-	-	-	6,5	0,36	3	6,0	0,44	2	37,5	4,36	2	-	-	-	-	-	-	-	-	-
LC	7,1	-	1	-	-	-	-	-	-	7,6	0,74	4	6,9	0,81	2	52,8	12,67	2	6,9	-	1	42,4	-	1	42,4	-	1
LP3	-	-	-	5,7	-	1	-	-	-	-	-	-	-	-	-	-	-	-	-	-	-	-	-	-	-	-	-
LP4	8,7	0,1	2	7,1	0,34	2	61,3	2,19	2	8,2	0,93	2	7,2	0,52	2	59,9	10,96	2	7,6	-	1	45,4	1,0	2	45,4	-	1
LM1	10,4	-	1	11,0	-	1	114,3	-	1	11,5	0,99	2	12,3	0,14	2	141,4	10,55	2	10,0	0,1	2	102,5	0,9	2	102,5	9,58	2
LM2	10,8	-	1	11,3	-	1	122,0	-	1	10,7	0,40	3	11,4	0,22	3	122,0	6,10	3	-	-	-	-	-	-	-	-	-
LM3	-	-	-	-	-	-	-	-	-	-	-	-	11,6	-	1	-	-	-	9,2	0,0	2	82,7	0,8	2	82,7	7,97	2

Supplementary Table S6: Number of Identified Specimens (NISP) by taxon along the stratigraphic sequence (data from Wilkens 1987) (see excel)

References

1 Giraudi, C. Lake levels and climate for the last 30,000 years in the Fucino area (Abruzzo-Central Italy)—A review. *Palaeogeogr. Palaeocl.* **70**, 249-260 (1989).

2 Grifoni Cremonesi, R. & Mallegni, F. Testimonianze di un culto ad incinerazione nel livello a ceramica impressa della Grotta Riparo Continenza di Trasacco (L'Aquila) e studio dei resti umani cremati. *Atti della Societa Toscana di Scienze Naturali* **85**, 253-279 (1978).

3 Grifoni Cremonesi, R. *Nuovi dati sul Mesolitico e sul Neolitico nella Piana del Fucino*. (Università di Roma La Sapienza, 1985).

4 Grifoni Cremonesi, R. La Grotta Continenza di Trasacco: note sui livelli epigravettiani. *Atti della XXXVI riunione scientifica: preistoria e protostoria dell'Abruzzo*, 81-89 (2003).

5 Grifoni Cremonesi, R. & Serradimigni, M. Grotta Continenza (Trasacco-AQ). *Rivista di Scienze Preistoriche* **LX**, 364-365 (2010).

6 Grifoni Cremonesi, R., Serradimigni, M. & Usala, M. Le campagne di scavo dal 2000 al 2009 nella Grotta Continenza presso Trasacco (AQ). *Atti del III Convegno di Archeologia e Il Fucino e le aree limitrofe nell'antichità*, 27-37 (2011).

7 Boschian, G., Serradimigni, M., Colombo, M., Ghislandi, S. & Cremonesi, R. G. Change fast or change slow? Late Glacial and Early Holocene cultures in a changing environment at Grotta Continenza, Central Italy. *Quatern. Int.* **450**, 186-208 (2017).

8 Antonio, M. L. *et al.* Ancient Rome: A genetic crossroads of Europe and the Mediterranean. *Science* **366**, 708-714 (2019).

9 Serradimigni, M. *et al.* Analyse spatiale des objets d'art mobilier du dépôt épigravettien de la Grotta Continenza (Abruzzes, Italie) et leur rapport avec les sépultures et les structures de combustion. *Bulletin de la Société Préhistorique Ariege-Pyrénées*, 65-66 (2012).

10 Wilkens, B. *Il passaggio dal Mesolitico al Neolitico attraverso lo studio delle faune di alcuni siti dell'Italia centro-meridionale* PhD thesis, Università degli studi di Pisa, (1987).

11 Wilkens, B. La fauna dei livelli neolitici della grotta Continenza. *Rivista di Scienze Preistoriche* **42**, 93-100 (1989).

12 Alhaique, F. Taphonomic analysis of the faunal remains from the "P" and "M" layers of the Arene Candide (Savona, Italy). *Quaternaria Nova* **4**, 263-295 (1994).

13 Compagnoni, B., Curci, A. & Tagliacozzo, A. in *Proceedings of the 7th ICAZ Conference (Konstanz, 26/09/1994-01/10/1994). Anthropozoologica.* 25-26.

14 Fiore, I. Lo sfruttamento dei mammiferi di piccola e media taglia nell'Epigravettiano di Grotta Romanelli. *Grotta Romanelli nel centenario della sua scoperta (1900-2000). Galatina: Congedo*, 137-148 (2003).

15 Alhaique, F. Strategie di occupazione nelle grotte del Fucino (Abruzzo) durante il Paleolitico superiore: il caso di Grotta Maritza (AQ). *Atti del 3° Convegno Nazionale di Archeozoologia*, 181-190 (2005).

16 Alhaique, F. in *L'adattamento umano all'ambiente, passato e presente. Atti XI congresso degli Antropologi Italiani. Abaco, Forlì.* 210.

17 Alhaique, F. archaeological deposits: the case of the Fucino Basin sites (Central Italy). *Archaeofauna* **12**, 61-71 (2003).

18 Fiore, I. & Tagliacozzo, A. Who ate whom? Carnivore tooth marks on animal bone remains in *13th ICAZ International Conference* (2018).

19 Wilkens, B. Animali da contesti rituali nella preistoria dell'Italia centro-meridionale. *Atti del 1° Convegno Nazionale di Archeozoologia*, 201-207 (1995).

20 Alhaique, F. & Recchi, A. Preliminary results of the re-analysis of the mammalian and avian remains from Grotta di Ortucchio (Avezzano). *Rivista di Antropologia* **79**, 73-79 (2001).
21 Barker, G. Prehistoric territories and economies in central Italy. *Palaeoeconomy. ES Higgs, ed* (1975).
22 Barker, G. *Landscape and society: prehistoric central Italy* (Academic Press, 1981).
23 Radmilli, A. M. *Gli insediamenti preistorici in Abruzzo* (Istituto Geografico Militare, 1959).
24 Radmilli, A. M. *La situazione degli studi paletnologici in Abruzzo* (1968).
25 Radmilli, A. M. *Considerazioni sul Mesolitico italiano*. (Universita degli studi, 1960).
26 Mussi, M. *et al. Tempi e modi del ripopolamento dell'Appennino centrale nel Tardiglaciale: nuove evidenze da Grotta di Pozzo (AQ)* (2008).
27 Bietti, A. Considerazioni sulla natura e sulla funzione sei siti del Tardiglaciale. *Istituto Italiano di Preistoria e Protostoria. Atti della XXXVI Riunione Scientifica: Preistoria e Protostoria dell'Abruzzo, Chieti–Celano, 27-30 settembre 2001*, 117-124 (2003).
